# Septin 9_i2 is downregulated in tumors, impairs cancer cell migration and alters subnuclear actin filaments

**DOI:** 10.1038/srep44976

**Published:** 2017-03-24

**Authors:** P. Verdier-Pinard, D. Salaun, H. Bouguenina, S. Shimada, M. Pophillat, S. Audebert, E. Agavnian, S. Coslet, E. Charafe-Jauffret, T. Tachibana, A. Badache

**Affiliations:** 1 Centre de Recherche en Cancérologie de Marseille, INSERM, Institut Paoli-Calmettes, Aix Marseille Univ, CNRS, Marseille, France; 2 Department of Bioengineering, Graduate School of Engineering, Osaka City University, Osaka, Japan

## Abstract

Functions of septin cytoskeletal polymers in tumorigenesis are still poorly defined. Their role in the regulation of cytokinesis and cell migration were proposed to contribute to cancer associated aneuploidy and metastasis. Overexpression of Septin 9 (Sept9) promotes migration of cancer cell lines. *SEPT9* mRNA and protein expression is increased in breast tumors compared to normal and peritumoral tissues and amplification of SEPT9 gene was positively correlated with breast tumor progression. However, the existence of multiple isoforms of Sept9 is a confounding factor in the analysis of Sept9 functions. In the present study, we analyze the protein expression of Sept9_i2, an uncharacterized isoform, in breast cancer cell lines and tumors and describe its specific impact on cancer cell migration and Sept9 cytoskeletal distribution. Collectively, our results showed that, contrary to Sept9_i1, Sept9_i2 did not support cancer cell migration, and induced a loss of subnuclear actin filaments. These effects were dependent on Sept9_i2 specific N-terminal sequence. Sept9_i2 was strongly down-regulated in breast tumors compared to normal mammary tissues. Thus our data indicate that Sept9_i2 is a negative regulator of breast tumorigenesis. We propose that Sept9 tumorigenic properties depend on the balance between Sept9_i1 and Sept9_i2 expression levels.

Actin filaments, microtubules and intermediate filaments are polymers constituting most of the cytoskeleton. Despite distinct structural and physical properties, crosstalk between these structures integrate mechano-transduction pathways and participate to cell shape plasticity. Septin filaments have been recently identified as an additional element of the cytoskeleton, interacting with microtubules, microfilaments and defined regions of the plasma membrane^1^. Septins are involved in cell division, a function conserved from yeast to humans, but also in the control of mammalian cell migration^2^. These processes are altered during tumorigenesis and participate to genetic instability and cancer cell dissemination at metastatic sites. Septins are GTP/GDP-binding proteins self-assembling in apolar hexamers or octamers, which can associate by their ends to form filaments in low salt conditions *in vitro*^3^,^4^. Yeast septin filaments can be paired via their coiled coil C-termini and even form gauze-like complex network of filaments^5^,^6^. In human carcinoma cell lines, septin filaments are recruited by anillin, in association with the actomyosin ring, to the cleavage furrow and to the intercellular microtubule bundle in late telophase during cytokinesis^7^,^8^. Septins also associate with actin filaments and microtubules in non-dividing cells^9^,^10^. Originally, human septins were cloned as fusion products with mixed lineage leukemia (MLL) gene^11^,^12^,^13^. Such MLL fusions resulting from translocations, usually involve oncogenic partners, but to date, MLL-septins oncogenic functions have not been clearly established^14^.

There are 13 human septins (Sept) distributed into four different groups (Sept2, Sept6, Sept7 and Sept3) based on sequence homology^15^,^16^,^17^. The structure of the human hexamer reveals the order in which septins associate with each other (Sept7::Sept6:Sept2::Sept2:Sept6::Sept7) and defines two alternate septin-septin interfaces, one between GTP/GDP binding domains (G:G) and one between N-termini/C-termini (NC::NC)^18^. Sept9 belongs to the Sept3 group; it associates to Sept7 and thus is positioned at the extremities of the Sept9:Sept7::Sept6:Sept2::Sept2:Sept6::Sept7:Sept9 octamers^19^,^20^. Sept9 has been repeatedly identified as a potential oncogene, and less frequently as a tumor suppressor^14^. SEPT9 gene, located at the Chr. 17q25 locus, is amplified in breast tumors with a positive correlation between amplification and tumor grade^21^,^22^. Sept9 expression is increased at the mRNA and protein levels in breast carcinomas, when compared to normal or peritumoral mammary tissues^22^. Similar trends were observed in the PyMT mammary carcinoma mouse model^22^. The SEPT9 gene could produce up to 18 alternatively spliced mRNA variants^23^. The expression of Sept9_i1 and Sept9_i3 has been reported to be frequently increased in breast tumors^21^,^22^,^24^. Several mechanisms may be operating to regulate the expression of septin isoforms during tumorigenesis, but a study showed that hypermethylation of DNA immediately upstream of the starting codon down-regulated Sept9_i3 expression^22^. Additional evidence of such epigenetic regulation of SEPT9 gene is detected reproducibly in the first exon coding for Sept9_i2 in colon adenoma^25^. Hypermethylation in this locus is detected in circulating DNA and is the base for a recently approved early detection assay of colon cancer^26^. These studies point towards a potential role of alterations in Sept9 isoforms expression profile during oncogenesis.

Despite the observation that Sept9_i1 interacts with microtubules^8^,^10^ and that Sept9_i3 is rather localized with actin filaments^8^,^27^, this has not been linked to a particular cellular function. In addition, whereas epigenetic studies on colon cancer suggest that Sept9_i2 expression could represent a marker of pre-cancerous status, Sept9_i2 expression has not been investigated at the protein level. In a previous study, preliminary results on the localization and function of GFP-Sept9_i2 construct in a breast cancer cell line suggested that this isoform could be distinguished functionally from Sept9_i1 and_i3^22^.

In the present work, we examined the contribution of specific Sept9 isoforms to breast cancer cell migration and further showed that in contrast to other Sept9 long isoforms, Sept9_i2 was not supportive of cancer cell migration. Moreover, Sept9_i2 expression altered the cellular distribution of septin associated actin filaments. Deletion of Sept9_i2 N-terminal specific sequence demonstrated that it is necessary for these differential effects. We produced a highly specific antibody against Sept9_i2 that we validated for immunohistochemistry. For the first time we showed that, the Sept9_i2 isoform is down-regulated in breast tumors of different types when compared to normal mammary tissue. Our results suggest that a change in the balance of Sept9 isoforms expression may be determinant for the evolution from a normal epithelium to a carcinoma with metastatic potential.

## Results

### *SEPT9_v2* first exon is hypermethylated in cancer

**Figure 1:**
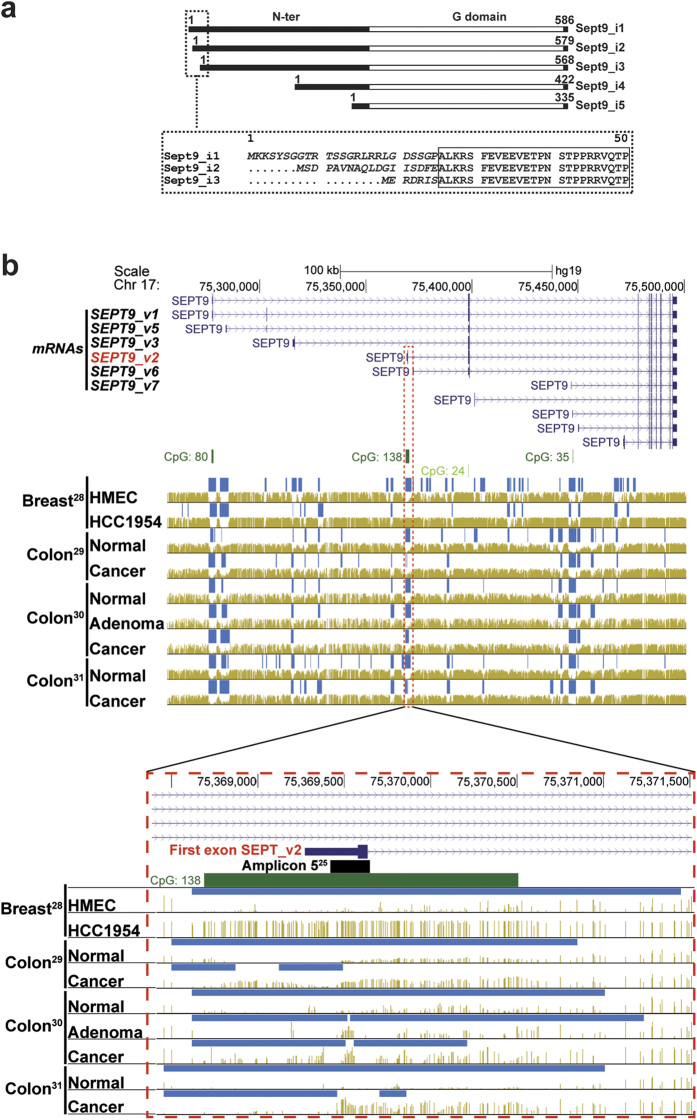
Differential CpG methylation levels of the SEPT9 gene in carcinoma vs normal epithelia. (**a**) Sept9 isoform proteins differ by their N-terminal domain; long isoforms, Sept9_i1,_i2,_i3 differ from each other by their starting amino acid sequences (indicated in italic and see Supplementary Fig. S1 for sequence details). (**b**) Top panel presents the human *SEPT9* gene locus on Chr 17 with 11 alternatively spliced mRNA variants having in common the last 9 exons; six of these transcripts are present in breast tissues^24^, *SEPT9_v1, _v2,_v3,_v5,_v6* and*_v7*, coding for Sept9_i1,_i2,_i3,_i4,_i4 and_i5 isoforms, respectively. Middle panel displays the position of CpG islands (dark green >300 bases; 200< light green <300 bases), hypo-methylated regions (dark blue bars on top of corresponding genome) and methylated CpGs (golden brown vertical lines whose height is proportional to CpG methylation level). Four groups of human methylome datasets are presented: first set, HMEC (Human Mammary Epithelial Cells, primary cell line) vs HCC1954 (grade 3, ductal carcinoma cell line)^28^; second set, normal colon and colon cancer derived primary epithelial cell lines^29^; third set, normal colon, colon adenoma and tumor biopsies^30^; fourth set, normal colon and colon cancer biopsies^31^. Bottom panel is the zoomed region delineated by a dashed red frame including the large CpG island 138 and the first exon of *SEPT9_v2*; the location of the differentially methylated amplicon identified by Wasserkort *et al*.^25^ in microdissected colon cancer cells is represented by a black bar on top of CpG 138 island (dark green bar). Public bisulfite-seq datasets were extracted from the MethBase methylome database using the UCSC Genome Browser on Human Feb. 2009 (GRCh37/hg19) assembly and the MethPipe software package (smithlabresearg.org).

Six among the 18 alternatively spliced *SEPT9* mRNA variants (*SEPT9_v1, _v2,_v3,_v5,_v6,_v7*) have been recently detected by quantitative RT-PCR in breast tissues^23^,^24^. The three long isoforms Sept9_i1,_i2 and*_*i3 differ by their extreme N-terminus (Fig. 1a) encoded by an alternative first exon (Fig. 1b). Remarkably, these first exons reside in genomic regions enriched in CpG^22^ (*SEPT9_v3*) and CpG islands^25^ (*SEPT9_v1* and *SEPT9_v2*) (Fig. 1b). Examination of CpG methylation levels, in genome wide high-resolution DNA methylation analyses^28^,^29^,^30^,^31^, revealed that the first exon of *SEPT9_v2* is hypermethylated at specific CpGs in a breast cancer cell line relative to normal breast epithelial primary cells and in colon cancer relative to normal colon. The data set including colon adenoma^30^ clearly showed that hypermethylation of *SEPT9_v2* first exon is induced in early development of colon tumors, and at a specific locus matching the hypermethylated amplicon 5 described independently by Wasserkort *et al*.^25^ (Fig. 1b). This preliminary examination of published datasets suggested that hypermethylation of *SEPT9_v2* first exon is a sensitive epigenetic marker of tumorigenesis, not only in colon, but also in breast cancer.

### Differential protein expression of Sept9_i2 is revealed by a highly specific monoclonal antibody

**Figure 2:**
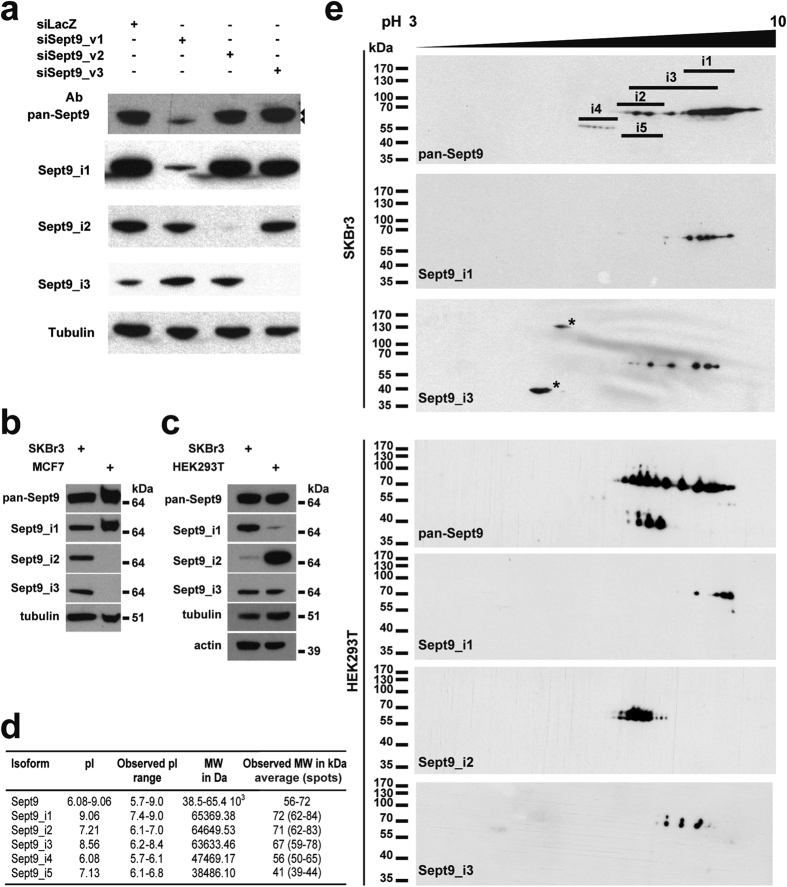
Highly specific monoclonal antibodies against Sept9_i1,_i2 and_i3 reveal differential expression profiles in cell lines. (**a**) SKBr3 cells were transfected with Sept9 isoform-specific siRNAs to eliminate individual endogenous isoforms. Western blotting using the different monoclonal antibodies directed against the extreme N-terminal sequence of Sept9_i1,_i2 or_i3 showed that the antibodies recognized only the targeted isoform. A polyclonal antibody directed against the C-terminus (pan-Sept9) common to all isoforms revealed that Sept9_i1 is the major isoform in SKBr3 cells. (**b**) Comparison of Sept9 long isoform expression in MCF7 and SKBr3 cells showed that only Sept9_i1 is expressed in MCF7 cells. Blots for each isoform were exposed for different durations in order to generate comparable signals. (**c**) Comparison of Sept9_i2 expression levels in HEK293T and SKBr3 cells by 1D Western blotting showed that Sept9_i2 and Sept9_i1 are strongly and weakly expressed in HEK293T cells, respectively. (**d**) Table presenting the calculated and observed MW and pI for each Sept9 isoform. (**e**) Proteins extracts of SKBr3 or HEK293T cell lines were separated by 2D-gel electrophoresis, and Western blotting was performed using the antibodies described in (**a**). The observed pI range for each Sept9 isoform is indicated by the horizontal bars in the upper blot. Sept9_i4 and_i5 spot assignments are solely based on apparent pI and MW revealed with the pan-C antibody. Asterisks indicate non-specific spots. The antibody used is indicated in the lower left corner of each blot. Blot images in panels (a–c) were cropped, full blot images are presented in Supplementary Fig. S8.

In order to analyze the expression of Sept9 isoforms in cell lines and tissues, we produced isoform specific rat monoclonal antibodies and analyzed Sept9 expression in various cell lines (Fig. 2). Antibodies were obtained using the extreme N-terminal sequence of Sept9_i1,_i2 or i3 (Fig. 1a and Supplementary Fig. S1) as antigens and were validated by different approaches. Expression of siRNAs designed to specifically target each of Sept9 long isoforms in SKBr3 breast cancer cells (a cell line, that expresses the three isoforms compared to MCF7 cells that expresses only Sept9_i1 (Fig. 2b)), revealed the specificity of the Sept9 isoform-targeting antibodies (Fig. 2a). Using a rabbit polyclonal antibody against the C-terminus common to all Sept9 isoforms (pan-Sept9), we observed that the siRNA against Sept9_i1 strongly decreased the expression of total Sept9 in SKBr3 cells, showing that Sept9_i1 is the major isoform in this cell line (Fig. 2a); the remaining electrophoretic band, that migrated slightly faster in SDS-PAGE than Sept9_i1 and_i2, corresponded most likely to Sept9_i3. Thus, we conclude that SKBr3 cells express Sept9 long isoforms in the following order of abundance: Sept9_i1 >> Sept9_i3 > Sept9_i2. In order to better characterize Sept9_i2 expression and further validate our antibodies, we searched for a cell line expressing Sept9_i2 at significantly higher levels than the SKBr3 cell line. We found that the embryonic HEK293T cell line was such a cell line (Fig. 2c). Sept9_i1,_i2 and i3 have different calculated isoelectric points (pI) (Fig. 2d); thus we performed bi-dimensional gel electrophoresis separation and analyzed endogenous Sept9 isoforms in HEK293T or SKBr3 cell lines by Western blotting (Fig. 2e). The Sept9 isoform-targeting antibodies detected series of spots and the most basic spot in each series had an apparent MW and pI closely matching the expected values of non-modified Sept9 isoforms (Fig. 2d). More acidic spots in each series were corresponding most likely to phosphorylated species with increased number of phosphorylated amino acid residues, suggesting yet another level of Sept9 isoforms regulation (Fig. 2e).

### Sept9_i2 is down-regulated in breast tumors

**Figure 3:**
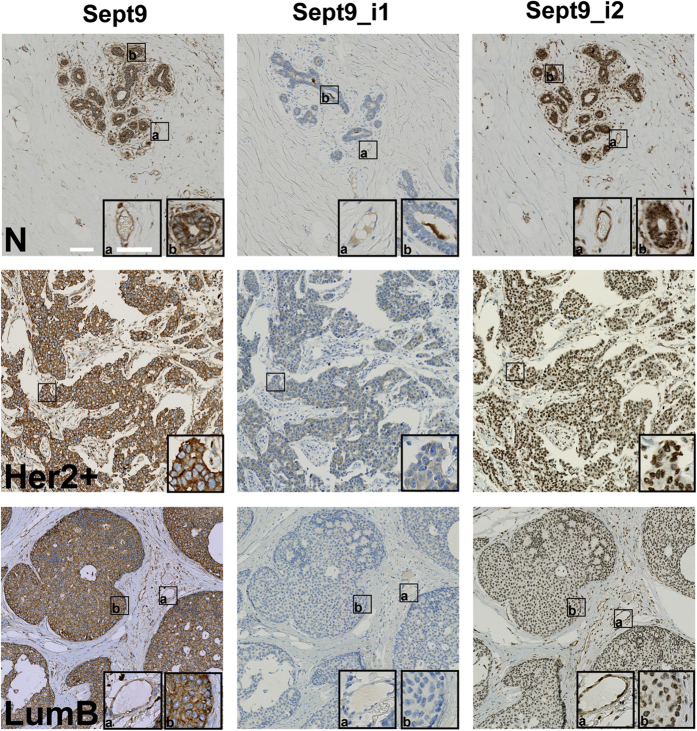
Expression of Sept9_ i2 is downregulated in breast tumors. Mammary gland tissues and breast tumors belonging to different sub-types were immunolabeled, as indicated, with a pan Sept9 antibody or antibodies specific for Sept9_i1 or for Sept9_i2, validated for immunohistochemistry (see Supplementary Fig. S2). Inserts correspond to zoomed boxed areas; (**a**) endothelial cell labeling, (**b**) epithelial and carcinoma cell labeling. Nuclei were stained blue. While both normal tissue and tumors are positive with pan-Sept9 antibodies, isoform-specific antibodies reveal differential expression. Sept9_i1 is increased in a subpopulation of tumors. An example of a positive and a negative tumor is presented in an Her2+ and LumB tumors, respectively. Sept9_i2 antibody shows strong cytosolic labeling in normal tissue only. Sept9_i2 nuclear staining observed in tumors was not specific (see Supplementary Fig. S2). N: normal; Her2+: ErbB2 overexpressing; LumB: luminal B. White horizontal bars represent 100 μm and 60 μm in images and zoom inserts, respectively.

By analogy to the effect of Sept9_i3 first exon hyper-methylation^22^, Sept9_i2 first exon hyper-methylation in tumors (Fig. 1b) could lead Sept9_i2 down-regulation in breast cancer. Therefore, we undertook the analysis of Sept9_i1 and_i2 protein expression in normal mammary gland tissue and tumors representative of breast cancer major subtypes (Table 1 and Fig. 3). We first validated the polyclonal pan-Sept9 antibody and Sept9_i1 and_i2-specific monoclonal antibodies for immunohistochemistry, using paraffin-embedded MCF7 cells which express only Sept9_i1 endogenously or MCF7 cells engineered to express only Sept9_i2 or i3 (Supplementary Fig. S2). Pan-Sept9 and Sept9_i1 antibodies generated strong cytoplasmic labeling in MCF7, which was abrogated upon Sept9_i1 knockdown (Supplementary Fig. S2); moreover the pan-Sept9 antibody showed a strong positive signal in Sept_i2 and Sept9_i3 expressing cells, while the Sept9_i1 antibody did not, illustrating the specificity of the antibodies. Finally, the anti-Sept9_i2 antibody yielded strong cytosolic staining only in Sept9_i2 expressing cells (Supplementary Fig. S2). Of note, the anti-Sept9_i2 antibody produced a non-specific nuclear staining that was revealed in Sept9_i2 negative samples.

Using the pan-Sept9 antibody, we observed virtually no difference in the expression of total Sept9 in normal breast epithelium vs. breast carcinoma (Table 1 and Fig. 3). Endothelial cells, present in normal and tumor sections, were positive for Sept9 expression (Fig. 3). Sept9_i1 was slightly over-expressed in tumors (Fig. 3, see Her2+ tumor example), with a higher incidence in basal subtype (Table 1). More strikingly, cytosolic expression of Sept9_i2 was clearly present in normal tissue, but not detected in the vast majority of tumors (the remainder staining observed in tumors corresponded to non-specific nuclear labeling); Sept9_i2 was expressed only in some tumors of the basal subtype, but at much lower levels than in normal breast tissues (Table 1). Isoform specific antibodies also revealed that endothelial cells expressed Sept9_i2 but not Sept9_i1 (Fig. 3). Thus, the use of isoform specific antibodies unveiled different patterns of Sept9 isoform expression and the strong down-regulation of Sept9_i2 in breast tumors, not detectable with pan-Sept9 antibodies.

### Overexpression of Sept9_i2 inhibits the migration of cancer cells

**Figure 4:**
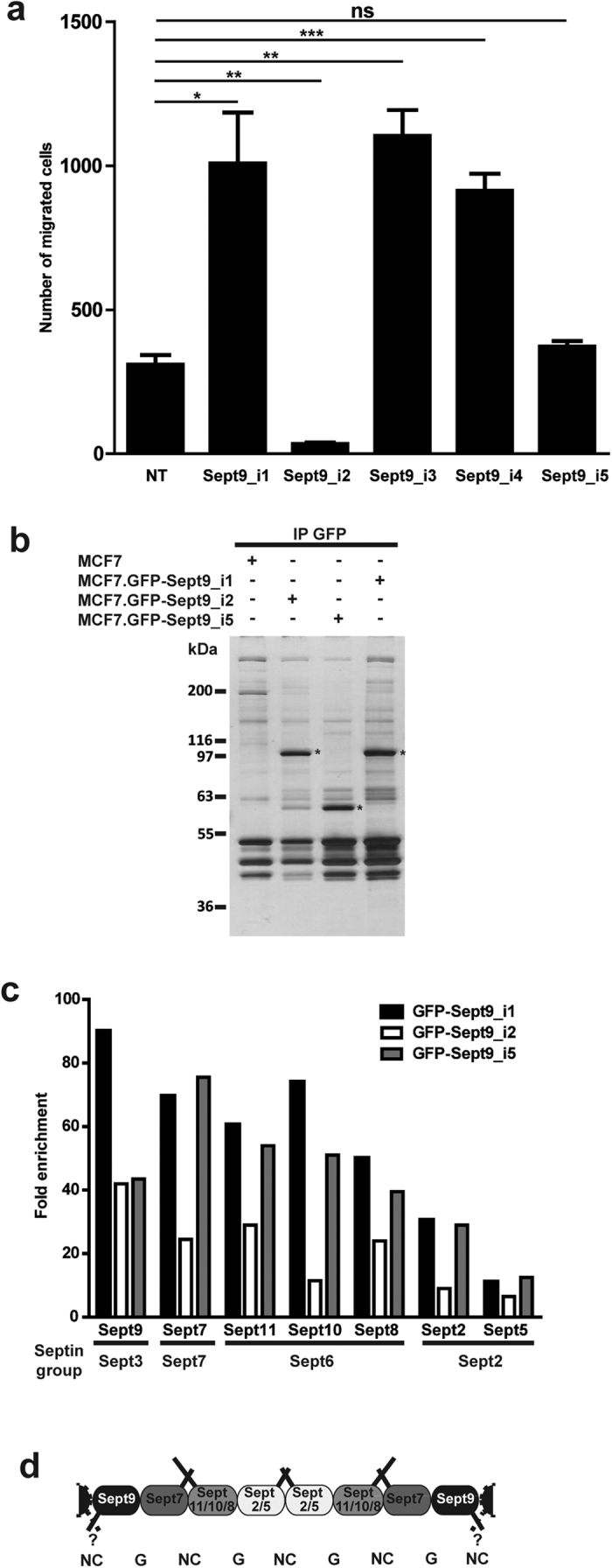
Sept9_i2 inhibits MCF7 cell migration. (**a**) Migration of MCF7 cell lines stably expressing one of Sept9 isoforms was measured in a transwell assay. The reference migrated cell number was of non-transfected (NT) MCF7 parental cell lines. Means and standard deviations from three independent experiments are presented; t-test, *p value < 0.05, **p value < 0.01, ***p value < 0.001. (**b**-**d**) Identification of the protein partners of specific Sept9 isoforms. (**b**) Silver stained SDS-PAGE analysis of GFP-pull down performed on extracts from MCF7 cells stably expressing either GFP-Sept9_i1 or_i2 or_i5, used for mass spectrometry analyses. Extracts from non-transfected MCF7 cells were used as a control. (**c**) Mass spectrometry analyses identified only other septin family members. Results are expressed as means of total ion intensity signal-based enrichment ratio of proteins in GFP-pulldowns relative to control from two independent experiments. Sept9-associated septins were assembled by septin groups. The average ratio for each septin was consistent with its position in the septin tetramer. The detailed results of two independent pull-downs are presented in Supplementary Table S1 and the Vulcano plots from one pull-down are presented in Supplementary Fig. S3. (**d**) Scheme of septin octameric polymer with the different septins identified in MCF7 cells with known G domain-G domain (G) and N-terminus/C-terminus-N-terminus/C-terminus (NC) interactions. Septin coil-coiled C-terminal interactions and Sept9 long N-termini putative interactions are represented as solid bars at the top and the bottom of the polymer, respectively.

SEPT9 gene methylation has the potential to regulate the expression of Sept9 isoforms and thus to generate different combinations of isoforms in cells. In order to assess if alteration in Sept9 isoforms expression profile has functional consequences, MCF7 cells stably expressing either GFP-Sept9_i1,_i2,_i3,_i4 or_i5 were generated^22^. Compared to MCF7 parental cell line, these cell lines overexpressed Sept9 with the same levels of endogenous Sept9 and similar levels of GFP-Sept9 constructs. While they grew at similar rates compared to the parental MCF7 cell line, all GFP-Sept9-expressing cell lines, except the Sept9_i2-expressing line, migrated significantly more rapidly in a transwell assay^22^. In the present study, we performed this migration assay after additional passages under geneticin selective pressure and with an additional starvation step prior to transwell migration induced by serum (Fig. 4a). We observed that GFP-Sept9_i1,_i3 and_i4 cells migrated with a three-fold increased rate compared to MCF7, whereas GFP-Sept9_i5-expressing cell migration was not different from control. Interestingly, in our hands, migration of GFP-Sept9_i2-expressing MCF7 cells was strongly reduced. Our results indicate that the Sept9 long N-terminal domain (Supplementary Fig. S1, up to the end of sequence in green) is required for a pro-migratory effect and that the short specific N-terminal sequence of Sept9_i2 could have anti-migratory effects.

### Sept9_i2 interactome analysis does not reveal differentially associated protein partners

The five Sept9 isoforms differ from each other by their N-terminus (Supplementary Fig. S1). The differential effects of septin 9 isoforms on cancer cell migration could be due to protein partners interacting differentially with their specific N-terminal region that might regulate septin assembly or use septins as a docking platform. Sept9 is present at each end of septin octamers and is supposed to interact with another Sept9 in the next octameric septin complex via an NC interface to form longer polymers; thus, the nature of Sept9 N-terminus may also influence the stability of this interface. To test these hypotheses, we performed GFP-trap pull-downs on MCF7.GFP-Sept9_i1,_i2 or_i5 cell extracts and identified Sept9-associated protein by label-free mass spectrometry (Fig. 4b–d, Supplementary Table S1 and Fig. S3). Surprisingly, we did not identify Sept9 isoforms specific proteins partners, but we identified co-precipitating septins belonging to other septin groups that were the same for all three Sept9 isoforms (Fig. 4c and d, and Supplementary Table S1 and Fig. S3). These results suggest that intrinsic characteristics of Sept9 isoforms may be sufficient to drive pro- or anti-migratory properties of septin octamers.

### Anti-migratory properties of Sept9_i2 depends on its specific N-terminal sequence

**Figure 5:**
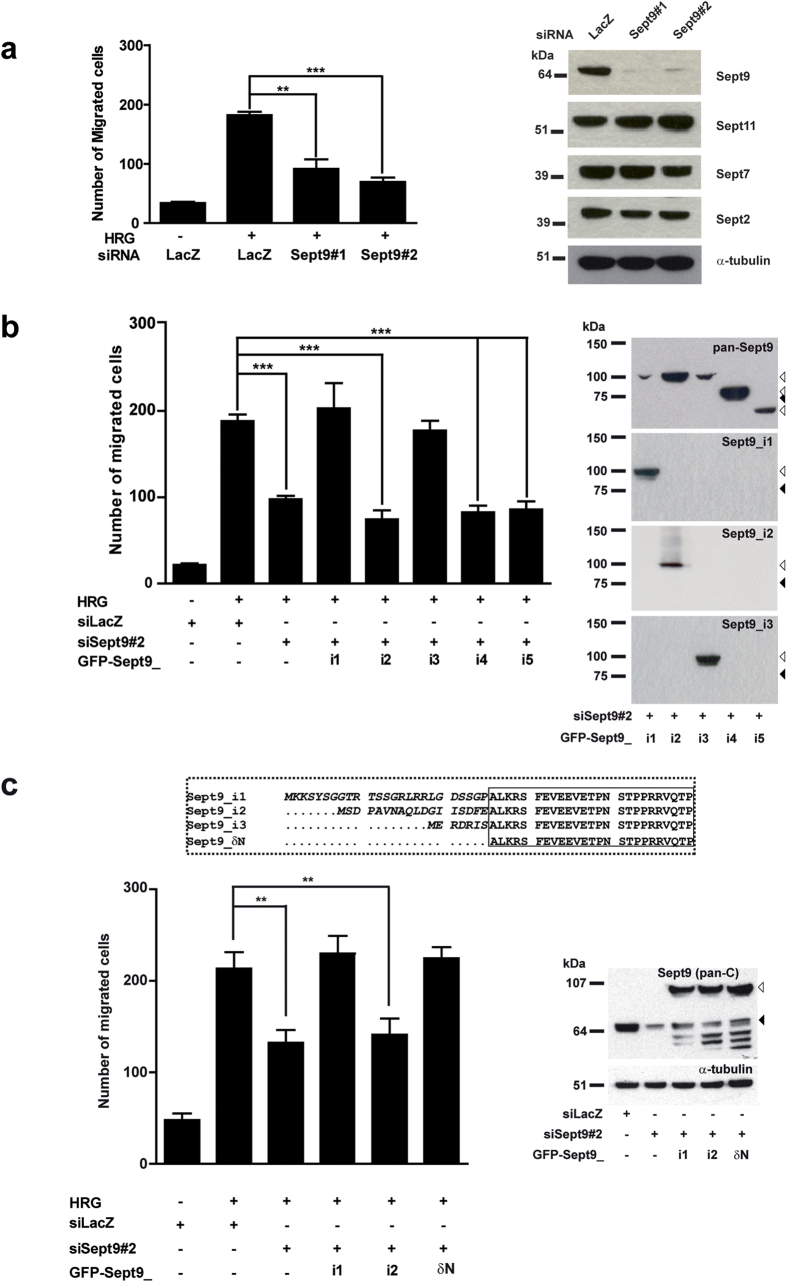
In contrast to Sept9_i1 and_i3, Sept9_i2 does not promote migration; role of its N-terminal sequence. (**a**) SKBr3 cells were transfected with control siRNA (LacZ), Sept9 siRNA#1 or #2 for 48 h before migration was measured in a transwell assay. SKBr3 cells express ErbB2 and their migration was induced by the presence of heregulin (HRG) in the bottom chamber. Sept9 knockdown inhibited HRG-induced cell migration. (**b**,**c**) SKBr3 cells were transfected with Sept9 siRNA#2 (directed against the 3′ UTR region) and cDNA coding for GFP-Sept9 isoforms or δN mutant. (**b**) Expression of Sept9_i1 and_i3, but not Sept9_i2, restored migration to control levels. (**c**) Expression of Sept9_ δN, deleted of the isoform specific sequence tag, restored migration to control levels. Results are from three independent experiments, mean ± S.D.; t-test, **p value < 0.01, ***p value < 0.001. The Western blots corresponding to each experimental condition presented on the left of the bar graphs confirm the efficiency of the siRNAs and the expression levels of the transfected constructs. White and black arrowheads indicate the gel motility of GFP-Sept9 constructs and endogenous Sept9, respectively. Blot images in panels a) were cropped, full blot images are presented in Supplementary Fig. S8.

The lower migration rate of MCF7 cells stably expressing Sept9_i2 (Fig. 4a) could be due to the selection of a poorly migrating cell population, independently of its Sept9 status. In order to confirm and extend our observations, we explored the impact of the various Sept9 isoforms on migration of a different cell model, the SKBr3 cell line. Migration of these ErbB2-expressing cells is strongly induced by addition of the growth factor heregulin (Fig. 5a). Down-regulation of Sept9 expression by two different siRNAs that targets all Sept9 isoforms (siSept9) -but did not affect the expression of other septins- decreased migration rate by an average of 50% (Fig. 5a). Upon transient expression of each GFP-Sept9 isoform construct in the presence of siSept9, we observed that Sept9_i1 and i_3 were the only isoforms able to restore migration to control levels (Fig. 5b). Sept9_i2 and Sept9_i5 did not rescue the defect in migration. These results confirm that Sept9 is involved in cancer cell migration and that Sept9_i2, contrary to the other two long isoforms, does not support migration. Surprisingly, contrary to its pro-migratory effect in MCF7 cells, Sept9_i4 could not restore migration in SKBr3 cells Sept9-depleted. This observation suggests that the N-terminal sequence of Sept9_i4 (Supplementary Fig. S1, sequence in green) was not sufficient to restore the migration rate to control levels.

Collectively, our results indicate that the variable extreme N-terminal sequence of long Sept9 isoforms influences cancer cell migration behavior. The distinctive effects of Sept9_i2 suggested that its specific N-terminal sequence impaired pro-migratory properties of Sept9. Therefore, we deleted Sept9_i2 N-terminus, producing Sept9_δN, a long Sept9 form with no specific extreme N-terminus (Fig. 5c). We observed that expression of Sept9_δN in SKBr3 cell knocked-down for Sept9 did restore migration to control levels (Fig. 5c) further confirming the critical role of Sept9_i2 N-terminus in the prevention of tumor cell migration.

### Sept9_i2 overexpression disrupts subnuclear septin associated actin filaments

Sept9 interacts with both microtubules and actin filaments and co-localizes with other septins in cells; thus, the differential effect of Sept9 isoforms on cell migration could be due to a differential interaction with and/or to an alteration of these cytoskeletal polymers. We observed differences in Sept9 isoform distribution in MCF7 cells stably expressing GFP-Sept9_i1,_i2,_i3,_i4 or_i5 (Supplementary Figs S4 and S5). Sept9_i5 interacted with neither microtubules nor actin filaments and displayed a diffuse labeling of the cytoplasm. Sept9_i1,_i2,_i3 and_i4 co-localized with actin filaments and Sept9_i1 was the only isoform associated with microtubules. Sept9_i1 being the only isoform expressed endogenously in MCF7 cells, we included a co-staining of total Sept9 to assess if there was an additional microtubule associated pool of Sept9 not labeled by GFP-Sept9_i2,_i3,_i4 or_i5 (Supplementary Figs S4 and S5). There was a perfect overlap between GFP and Sept9 labeling, strongly indicating that no such pool was present in these MCF7 cell lines. Thus, exogenous expression of Sept9 isoforms, other than Sept9_i1, removed endogenous Sept9_i1 from microtubules. Sept9_i2 co-localized with microfilaments, but septin filaments appeared shorter and disorganized compared to those in Sept9_i3 expressing cells (Supplementary Fig. S5). Interestingly, GFP-Sept9_i4 was also incorporated in short filaments associated with actin filaments (Supplementary Fig. S4) that, in contrast with the more dispersed GFP-Sept9_i2 filaments, were concentrated towards the center of the cell (Supplementary Figs S4 and S5).

**Figure 6:**
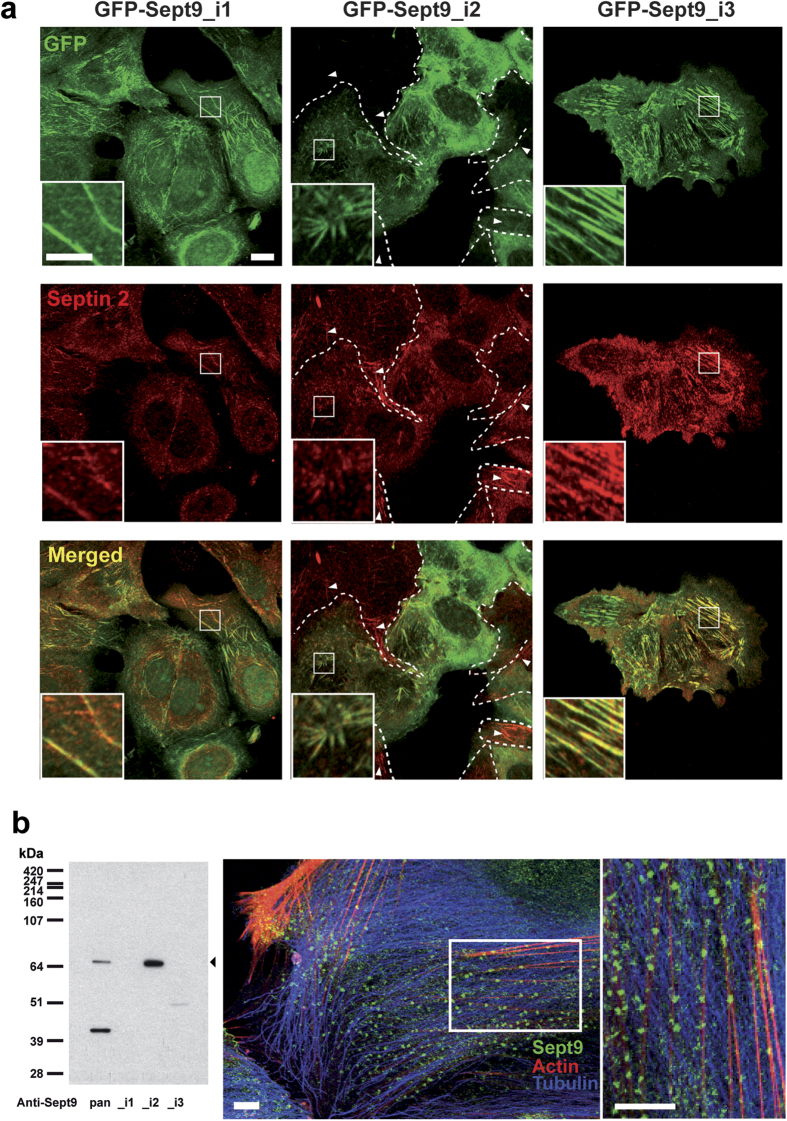
Sept9_i2 is incorporated into short septin filaments. (**a**) Septin 2 and 9 co-localization indicative of septin polymers was assessed in in MCF7 cells stably expressing Sept9_i1,_i2 or_i3. GFP-Sept9_i1 was incorporated in long Sept2-filaments (mainly aligned with microtubules as depicted in Supplementary Fig. S4). GFP-Sept9_i2 was incorporated in very short and disorganized Sept2-filaments (aligned with actin fibers as depicted in Supplementary Fig. S5). GFP-Sept9_i3 was incorporated in long Sept2-filaments (aligned with actin fibers as depicted in Supplementary Fig. S5). White arrowheads point to septin filaments in cells negative for GFP-Sept9_i2 expression (delimited by dashed white lines). Framed regions are zoomed at the bottom of each image. White bars correspond to 10 μm and 5 μm in image and zoomed regions, respectively. (**b**) HUVEC cells, which expressed only the Sept9_i2 long isoform as indicated by Western blotting (left panel). Immunolabeling with pan-Sept9 antibody showed that Sept9_i2 was concentrated in very small clusters associated with actin stress fibers. White bars correspond to 10 μm.

Because Sept2 and Sept9 occupy central and end positions in septin octamers, we co-stained these MCF7 cell lines for Sept2 (Fig. 6a and Supplemental Fig. S6). As expected, Sept2 co-localized with GFP-Sept9_i1 and_i3 in long filaments (Fig. 6a). In contrast, GFP-Sept9_i2 and Sept2 co-localized in short filaments (Fig. 6a). GFP-Sept9_i2 expressing MCF7 cell population was heterogeneous with some cells clearly expressing high and other expressing low or undetectable levels of GFP-Sept9_i2 (Fig. 6a, delimited by dashed white lines). In the former population, Sept2 was incorporated into GFP-Sept9_i2 short filaments, whereas in the latter subpopulation, Sept2 was incorporated into long filaments (Fig. 6a, white arrowheads). Interestingly, in primary endothelial cells (HUVEC), which expressed only the long Sept9_i2 isoform, Sept9 was incorporated in very small clusters distributed along actin stress fibers (Fig. 6b). Collectively, these results suggest that Sept9_i2 incorporation in septin octamers favors the formation of a population of short septin filaments associated with actin filaments. Sept2 and GFP-Sept9_i4 co-localized in short filaments that were specifically associated with the nuclear region (Supplementary Fig. S6).

**Figure 7:**
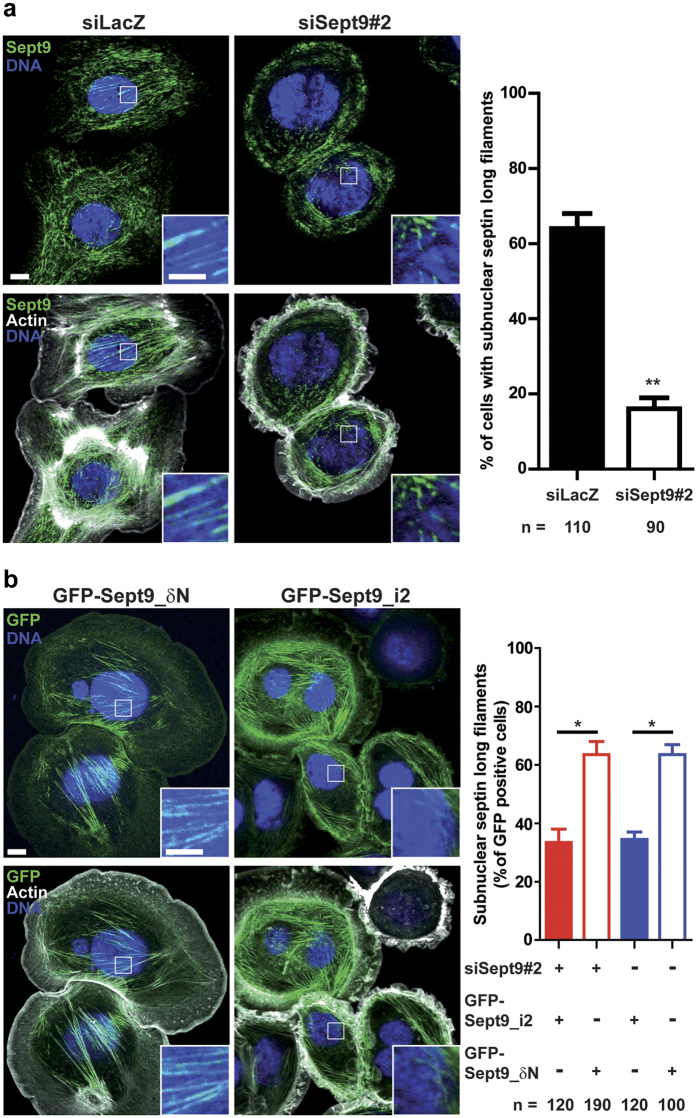
Sept9_i2 overexpression induces a loss of septin associated actin filament that is dependent on its N-terminal sequence. (**a**) SKBr3 cells were transfected either with control siRNA or siRNA targeting Sept9#2. After Sept9 knockdown, the number of cells with subnuclear long septin filaments associated with actin fibers decreased significantly as shown in the bar graph. Results are from the observation of the number of cells indicated at the bottom of the graph and collected in a duplicate, mean ± S.D.; t-test, **p value < 0.01. (**b**) SKBr3 cells were transfected either with GFP-Sept9_δN or GFP-Sept9_i2, and with or without the Sept9#2 siRNA. The number of cells with subnuclear long septin filaments associated with actin fibers decreased significantly when GFP- Sept9_δN was present. Results are from the observation of the number of cells indicated at the bottom of the graph and collected in a duplicate, mean ± S.D.; t-test, *p value < 0.05. Image panel display cells representative of cells expressing either GFP-Sept9_δN or GFP-Sept9_i2. Framed regions are zoomed at the bottom of each image. White bars correspond to 10 μm and 5 μm in image and zoomed regions, respectively.

In SKBr3 cells where Sept9 was knocked-down, we observed that the remainder Sept9 was associated with actin filaments located mainly out of the nuclear region (Fig. 7a). In contrast, subnuclear long septin filaments associated to actin filaments were observed in more than 60% of control cells. We wondered if SKBr3 cells overexpressing Sept9_i2 had similar alterations of subnuclear septin filaments, which would suggest that subnuclear septin long filaments are required for cell migration. Indeed, Sept9_i2 overexpression in SKBr3 cells knocked-down for Sept9 reduced the percentage of cells with subnuclear septin long filaments to 35% (bar graph of Fig.7b). In contrast, overexpression of Sept9_δN restored this percentage to more than 60% of GFP-positive cells (bar graph of Fig. 7b). We observed the same difference in % of GFP-positive with subnuclear septin long filaments between Sept9_i2 and Sept9_δN overexpressing cells when endogenous Sept9 was not knocked-down (Fig. 7b). Thus, Sept9_i2 had a dominant negative effect on subnuclear septin long filaments.

Collectively, our results strongly suggest that long subnuclear septin associated actin filaments support cell migration and that the specific N-terminus of Sept9_i2 is involved in their disruption by Sept9_i2.

### Sept9_i1 associates with acetylated microtubules

**Figure 8:**
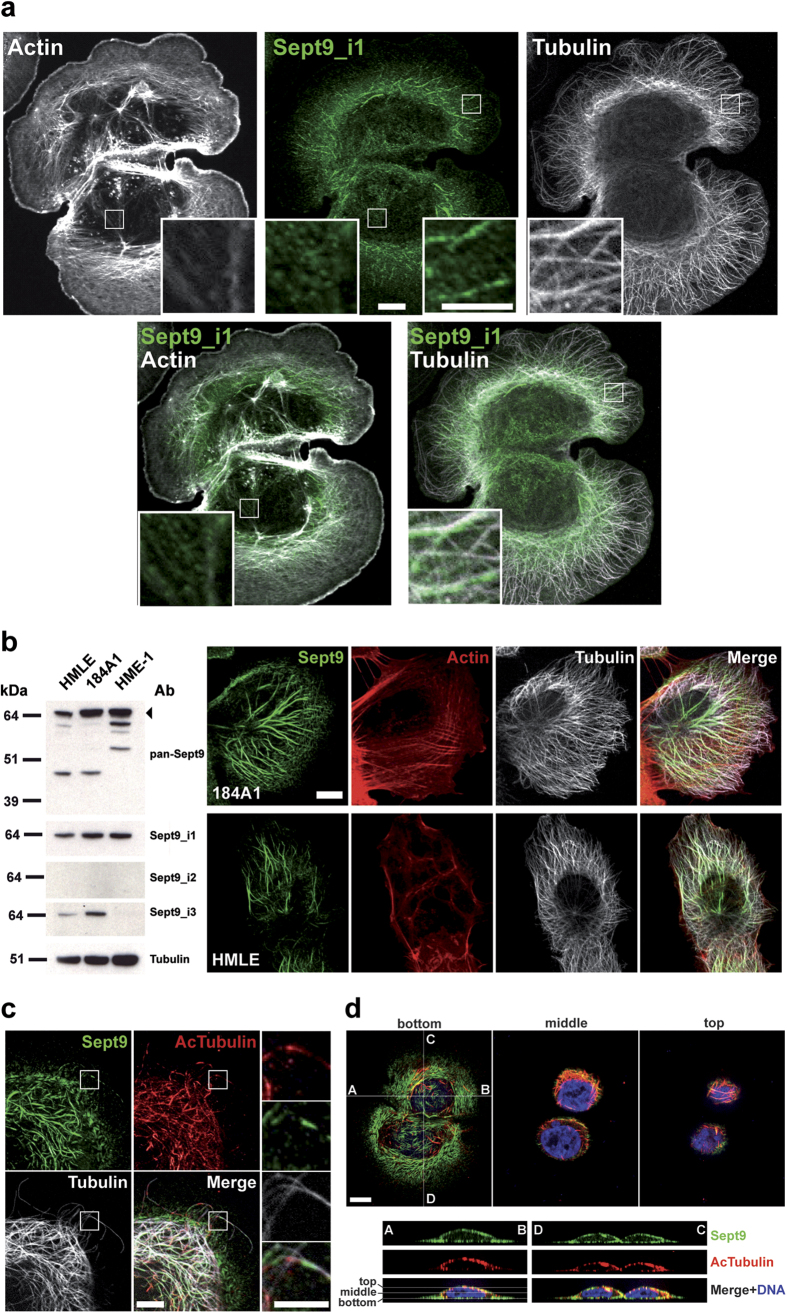
Sept9_i1 associates with a subpopulation of microtubules that are acetylated. (**a**) Labeling of SKBr3 cells with the anti Sept9_i1 antibody revealed that Sept9_i1 associated with a subpopulation of microtubules (right panels). A weaker labeling of microfilaments was also detected (left panels). Framed regions are zoomed at the bottom of each image. White bars correspond to 10 μm. (**b**) In immortalized human mammary epithelial cells that expressed the Sept9_i1 long isoform, but no Sept9_i2, Sept9 co-localized mainly with a subpopulation of microtubules oriented towards the leading edge. White bar corresponds to 10 μm. Blot images in panel b) were cropped, full blot images are presented in Supplementary Fig. S8. (**c**) Sept9 associated with deacetylated regions of acetylated microtubules in SKBr3 cells. Uncropped image of this cell and fluorescence intensity profiles of Sept9 vs acetylated tubulin along microtubules showing alternative patterns of labeling are presented in Supplementary Fig. S7. Framed regions are zoomed of the right side of the panel. White bars correspond to 10 μm and 5 μm in image and zoomed regions, respectively. (**d**) Acetylated and Sept9 labeled microtubules were mostly located on the edge and on top of the nucleus and Sept9 was present also under the nucleus (aligned with actin fibers as depicted in Fig. 7). Z-sections along the indicated A-B and D-C axes are presented at the bottom. White bar corresponds to 10 μm.

In SKBr3 cells, endogenous Sept9_i1 localized on a subpopulation of microtubules associated with the large protusion forming the leading edge of migrating cells in the presence of heregulin (Fig. 8a). A much weaker staining of actin filaments was also observed at the rear of these cells (Fig. 8a). In immortalized normal breast epithelial cell lines expressing mostly Sept9_i1, we observed even more clearly such a subpopulation of microtubules decorated by Sept9 in polarized cells (Fig. 8b). Orientation of microtubules with increased stability towards the leading edge of migrating cells has been observed in several model cell lines (see discussion). Acetylation of tubulin is a robust marker of stable microtubules, thus we performed co-staining of Sept9 and acetylated tubulin in SKBr3 cells (Fig. 8c). Close examination of microtubules at the periphery of the cells revealed that Sept9 associated with acetylated microtubules in an alternative pattern, where Sept9 associated with portions of these microtubules harboring lower levels of acetylation (Fig. 8c and Supplementary Fig. S7). Co-localization of Sept9 with acetylated microtubules was observed in cell protusion, but was mostly concentrated at the edge and the top of the nucleus. Sept9 was also present in filaments under the nucleus (Fig. 8d).

### Sept9 long isoforms co-sediment differentially with microtubules and F-actin

**Figure 9:**
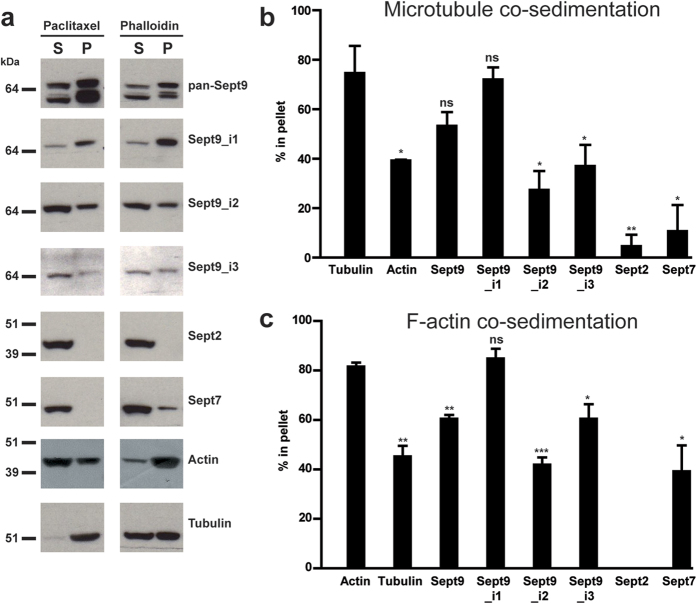
Differential interaction of endogenous Sept9 isoforms with microtubules and F-actin. (**a**) SKBr3 cell lysates were incubated with either paclitaxel or phalloidin to polymerize tubulin or G-actin, respectively. After high-speed centrifugation through a sucrose cushion, supernatant and pellet proteins were separated and equivalent amounts of each fraction were analyzed by Western blotting with the indicated antibodies and distribution of each protein in pellets (P) and supernatants (S) was quantified. The percentage of each analyzed protein in the pellets was compared to (**b**) the percentage of tubulin present in the paclitaxel-microtubule pellets or to (**c**) the percentage of actin present in the phalloidin-F-actin pellets. Results are from three independent experiments, mean ± S.D.; t-test, *p value < 0.05, **p value < 0.01, ***p value < 0.001. Blot images in panels a) were cropped, full blot images are presented in Supplementary Fig. S8.

Sept9_i1, because of its dual interaction with microtubules and actin filaments and being the major isoform expressed in cell lines such as SKBr3, contributes strongly to the complexity of Sept9 filament networks in these cells. We implemented a biochemical assay on cell lysates to determine if endogenous Sept9 isoforms co-sedimented differentially with microtubules whose polymerization and stabilization was induced by paclitaxel (Fig. 9a,b) and with F-actin whose polymerization was induced by phalloidin (Fig. 9a,c). Sept9_i1 was the only isoform to sediment as efficiently as tubulin and actin present in paclitaxel-microtubules and phalloidin-F-actin pellets, respectively. This result is coherent with the localization of Sept9_i1 with both microtubules and actin filaments observed in SKBr3 cells (Fig. 8a) and in MCF7 cells (Supplementary Figs S4 and S5). In addition, Sept7, a direct interactor of Sept9, and Sept2 were poorly co-sedimenting with tubulin and actin present in paclitaxel-microtubules and phalloidin-F-actin pellets, respectively. These results suggests that in the present assay, co-sedimentation was independent of septin octamers and was competitively dominated by the major Sept9 isoform expressed, Sept9_i1, interacting directly with both microtubules and actin filaments.

## Discussion

The hypermethylation of *SEPT9_v2* first coding exon in the circulating DNA from colon adenoma and carcinoma patients indicates a potential role for *SEPT9_v2* gene regulation in oncogenesis^25^. Analysis of high-resolution methylome data sets confirmed that, indeed, methylation of a specific amplicon in the CpG island containing the first coding exon for Sept9_i2 is significantly increased in colon adenoma and carcinoma. Interestingly, this CpG island is also hypermethylated in a breast cancer cell line compared to a primary normal mammary epithelial cell line, but the consequence on Sept9_i2 expression at the protein level is not known. Nonetheless, our results showing a systematic down-regulation of Sept9_i2 protein expression in breast tumors relative to normal mammary gland suggest that this particular locus might be also hypermethylated in breast tumors. Hypermethylation of DNA within promoters is generally associated with a down-regulation of the gene^32^. A previous study demonstrated that hypermethylation of a small region, including the TSS site, upstream of the start codon for Sept9_i3 down-regulates its expression^25^. This region overlaps with the first exon of Sept9_i3, indicating that the methylation status of the first exon of Sept9 isoforms could determine their relative expression. Accordingly, the apparent decrease in methylation in regions surrounding the first exon coding for Sept9_i1 (Fig. 1) may enable the expression of this isoform in carcinoma. Further combined analyses of SEPT9 gene methylation, mRNA and protein expression in different tissues and tumors is required to establish the robustness of the relationship between local DNA methylation and expression of specific isoforms. Alternative splicing produces the different mRNAs encoding the septin 9 isoforms. Recently, investigations on the role of DNA methylation in alternative splicing have identified mechanisms whereby the first exon of a transcript is preferentially recruited when hypomethylated^33^. We speculate that the level of methylation in first exons of Sept9 isoforms regulates alternative splicing.

The down-regulation of Sept9_i2 in breast tumors indicated the possible loss of a tumor suppressive function. Of note, the deletion of SEPT9 gene was previously observed in ovarian tumors^34^. Similarly, the MLL-SEPT9 fusion identified in certain leukemia^11^ could induce a loss of Sept9 function. This is also coherent with the decrease in total Sept9 expression observed in colon adenoma and tumors where Sept9_i2 first exon is hypermethylated^25^,^35^ suggesting that the loss of Sept9_i2 expression was apparently not compensated by an increase in the expression of other isoforms. Immortalized mammary epithelial cell lines with no tumorigenic potential did not express Sept9_i2, but expressed high Sept9_i1 levels instead (Fig. 8b). This suggests that the expression of Sept9_i1 alone is not sufficient for conferring oncogenic properties to cells. We speculate that the down-regulation of Sept9_i2 or the expression of Sept9_i1 at higher level than Sept9_i2 potentiates cells for acquiring oncogenic properties. In relation with the present study, changes in the balance of expression of Sept9 isoforms would affect migratory potential of tumor cells. How does Sept9_i2 expression could inhibit cell migration? We observed that overexpression of Sept9_i2 specifically induced a loss of long septin filaments associated to actin cables under the nucleus, which was strongly correlated with the inhibition of breast cancer cell migration in a transwell assay. Our observations point to a role of specialized subnuclear actin cables during cell migration whose presence could be regulated by the Sept9 isoform expression profile. Such subnuclear (2D conditions) and perinuclear (3D conditions) actin cables have been recently described as being nucleated by the formin FMN2 whose down-regulation induces a loss of these actin cables, inhibits 3D migration, and inhibits extravasion and lung metastasis of melanoma cells *in vivo*^36^. The pro-migratory effect of long septin filaments associated to actin filaments is not limited to cancer cells in tumors and a recent study showed that over-expression of CEP3 (Cdc42 Effector Protein 3 or Borg2), a regulator of septins, induced the assembly of small septin polymers in longer filaments associated with stress fibers, which transformed normal fibroblasts in the more motile and invasive cancer-associated fibroblasts (CAFs)^37^. In addition, a recent study suggested that epithelial cell motility is dependent on the cross-linking of stress fibers by septin filaments in the lamella which would regulate the dynamics of focal adhesions^38^. Therefore, different pathways (overexpression of CEP3 and the down-regulation of Sept9_i2) that promote the reorganization of septins in long polymers associated with actin filaments would favor cell motility in tumors and metastasis.

Further studies are required to characterize the mechanism underlying the distinctive effects of Sept9 isoforms on carcinoma cell migration, but it seems to involve long septin associated actin filaments within the nuclear region. However, microtubules are also known to regulate important aspects of cell migration, such as polarity^39^ and chemotaxis^40^,^41^,^42^. And regulation of tubulin acetylation on a sub-population of microtubules contributes to cancer migration and invasion^43^,^44^,^45^. Consistent with observations by others, we showed that Sept9_i1 is the only isoform to associate with microtubules, and that Sept9_i3 binds only to actin filaments^8^,^10^,^27^. We noticed that Sept9 labeling was coincident with deacetylated regions of acetylated microtubules. This observation is consistent with Kinoshita and co-workers findings showing that septin polymers are required for docking the tubulin-targeting deacetylase HDAC6 on microtubules thereby locally facilitating deacetylation of microtubules^46^. Sept9_i1 associates with both microtubules and actin filaments, thus the increase in Sept9_i1 may promote cancer cell migration by spatially organizing subpopulation of actin filaments and microtubules, such as the subnuclear actin cables described above, and acetylated microtubules oriented towards the leading edge.

Our data bring novel insights into the mode of association of Sept9 to specific cytoskeleton elements. The long structurally disordered N-terminal domain shared by all three long Sept9 isoforms was initially proposed to mediate Sept9 binding to, and bundling of microtubules^47^,^48^. Mutations of the basic residues in the imperfect repeats, located within the first basic half of the Sept9 N-terminus (Supplementary Fig. S1), abrogated the binding to microtubules^47^. It was suggested that, by analogy to basic repeats found in microtubule binding domains of MAPs^49^, Sept9 binds to the acidic C-terminus of tubulins located at the surface of microtubules^47^. However, recent data show that Sept9 basic region of its N-terminus can also bind and bundle F-actin *in vitro*; one mode of interaction involves amino-acid residues 1–7 of actin^48^, an acidic sequence accessible on the surface of F-actin. Therefore, the basic region of Sept9 N-terminus would not target specifically Sept9 to a specific cytoskeletal element, but may participate in the binding to both microtubules and actin filaments.

Our own examination of Sept9 N-terminal sequence revealed potential secondary structures in the form of short β-strands (Supplementary Fig. S1). Interestingly, this predicted local folding sites matched four of the five repeats we identified (Supplementary Fig. S1). One repeat is of particular interest because it is the site of two SEPT9 mutations found in hereditary neuralgic amyotrophy (HNA) patients^50^. The fact that R88W (reference sequence is SEPT9_i3) decreased microtubule bundling -but not binding- to microtubules^47^ was hypothesized as an underlying mechanism for the loss of neurite asymmetric growth in PC12 cells, and consequently for HNA^47^. This is not intuitively coherent with the existence of HNA cases where no point mutations, but a duplication of the region including repeats are found in SEPT9^51^, which would indicate a gain rather than a loss of a function. In any case, these repeats are common to all septin 9 long isoforms and therefore could not explain their differential localization in cells. In contrast, the tag formed by the short N-terminal sequence specific of each long isoform appears to address Sept9-containing septin polymers to microtubules and/or actin filaments. Noticeably, the Sept9_δN mutant, deprived of the specificity of each long isoform, localized in long septin filaments associated with actin filaments like Sept9_i3 did. The Sept9_i2 isoform sequence tag disorganized such filaments and the Sept9_i1 sequence tag targets septins to microtubules in addition to actin filaments. Structural analysis of Sept9 isoforms and *in vitro* reconstitution experiments with septin octamers containing specific Sept9 isoforms will be required to determine how the highly disordered N-terminus, repeats and specific extreme N-termini fine-tune interactions with septin assemblies and association with the cytoskeleton. Post-translational modifications (PTM) of Sept9 are concentrated in the N-terminus (www.phosphosite.org) and this could add another layer of regulation of Sept9 localizations and functions. Indeed, our 2D-PAGE analysis of Sept9 revealed the existence of several PTM whose profile may vary in different cell lines and culture conditions. These hypotheses are supported by a study showing that phosphorylation of Sept9 N-terminus by Cdk1 induces a Pin1-dependent change in its conformation that is required for the completion of abscission^52^. We speculate that the short N-terminal specific sequence of long isoforms is interacting with the septin polymer itself to create different surface topologies interfacing preferentially with microtubules or microfilaments, and/or with differential affinities for other septin octamers.

Our data show that Sept9 i_2 is downregulated in breast tumors and alters actin filament subcellular distribution and cancer cell motility when overexpressed. Thus, further characterization of the function of Sept9_i2 in normal epithelia and cells of the tumor micro-environment is crucial for both consolidating the role of septins in the transition to cancer, and validating Sept9 isoform expression profiling as a useful biomarker for early cancer detection.

## Methods

### Cell lines and culture

MCF7 and SKBr3 cell lines were purchased from ATCC; Cristina Montagna (AECOM, NY) kindly provided MCF7 cell lines stably expressing GFP-Sept 9 isoforms. MCF7, SKBr3 and HEK293T cell lines were cultured in DMEM (Gibco) with 10% fetal bovine serum (FBS), supplemented with geneticin for GFP-Sept9 expressing MCF7 cell lines. Human umbilical vein endothelial cells (HUVEC), isolated as previously reported by Kaplanski *et al*.^53^ and used between passages 2 and 4, were kindly provided by Michel Aurrand-Lions (CRCM, Marseille, France). HUVEC were cultured in complete EGM2 (Promocell) on 0.1% gelatin and 5 ng/ml fibronectin pre-coated petri-dishes. Immortalized normal human mammary epithelial cell lines 184A1, HMLE and HME1 were a kind gift from Christophe Ginestier (CRCM, Marseille, France). They were cultured in DMEM/F12 (Gibco) supplemented with 5% (184A1) or 10% (HMLE) horse serum or 0.1% BSA (HME1), 1% NEAA, 10 μg/ml insulin (Actrapid, Novo Nordisk), 1 μg/ml hydrocortisone, 10 ng/ml EGF, and 0.1 μg/ml cholera toxin (184A1 and HME1). All cell lines were culture at 37 °C in humidified 5% CO2 atmosphere.

### Human breast tissues

All samples of human origin and associated data were obtained from the IPC/CRCM Tumour Bank that operates under authorization # AC-2007-33 granted by the French Ministry of Research for tumor sample. Before scientific use of samples and data, patients were appropriately informed and asked to consent in writing, in compliance with French and European regulations. The project was approved by the IPC Institutional Review Board.

### Antibodies

The pan Sept9 polyclonal antibody against Sept9 C-terminus was a kind gift from Pascale Cossart (Institut Pasteur, Paris, France). We produced rat monoclonal antibodies against Sept9_i1, i2 or i3 specific N-termini (Supplementary Fig. S1) using the rat medial iliac lymph node method as described previously^54^. The following commercial antibodies were used for Western blotting (WB) and/or immunofluorescence (IF): polyclonal anti-Sept2 from Sigma (IF, Prestige Antibodies), polyclonal anti-Sept2 from Millipore (WB), polyclonal anti-Sept7 and anti-Sept11 (WB, Sigma), monoclonal anti-α-tubulin (DM1A, WB/IF, Sigma), monoclonal anti-acetylated tubulin (6-11B1, IF, Sigma), monoclonal anti-actin (AC-15, WB, Sigma). Phalloidin TRITC was from Sigma. AlexaFluor 594, AlexaFluor 488 and DyLight 405 secondary antibodies were from Jackson ImmunoResearch.

### siRNAs and plasmids

siRNAs targeting the 3′UTR or coding sequences of septin 9 were purchased from Life Technologies. The following siRNA were used: Sept9#1 (5′-GCACGAUAUUGAGGAGAAATT-3′) targeting the coding region and Sept9#2 (5′-GGAUCUGAUUGAGGAUAAATT-3′) targeting the 3′UTR of all SEPT9 mRNA variants; *SEPT9_v1* (5′-GCACCAUGAAGAAGUCUUATT-3′), *SEPT9_v2* (5′-GCAGCUGGAUGGGAUCAUUTT-3′), *SEPT9_v3* (5′-GCUGCUAAAUAUAUCCGUATT-3′) targeting the specific isoforms; a control siRNA against LacZ (5′-GCGGCUGCCGGAAUUUACCTT-3′) was used. Cristina Montagna (AECOM, NY) kindly provided N-terminally GFP-tagged septin 9 isoform constructs cloned in pEGFP-C2. GFP-SEPT9_v1 was used a matrix to generate the septin 9_δN mutant by PCR. siRNA were transfected using lipofectamine RNAiMAX (Invitrogen); siRNAs plus plasmids were transfected using AMAXA cell line nucleofector kit V (Lonza), following manufacturer’s instructions.

### Boyden chamber assay

Heregulin-induced SKBr3 cell migration was performed in 8-μm pore membrane Transwell chambers (Corning Costar), as described previously^55^, except that nuclei of migrated cells were labeled with 5 μg/ml Hoechst 33342 (Sigma) in water. For MCF7 cell lines, the same protocol was followed except that 10% FBS was used as chemoattractant. The entire bottom face of the Transwell insert membrane was imaged and cell counted with the cell counter function of Image J software on ten squares of 93625 pixel^2^ each.

### GFP pull-down and analysis by mass spectrometry

GFP-Sept9 expressing MCF7 cell lines were lysed and GFP pulldowns performed as described previously^53^ using GFP-Trap agarose beads (Chromotek, Planegg-Martinsried, Germany). Control pull-downs were obtained from lysates of parental MCF7. Mass spectrometry analysis was performed as described previously^56^ on two independent series of pull-downs using an LTQ-Orbitrap-Velos (Thermo Electron, Bremen, Germany) online with a nanoLC Ultimate 3000 chromatography system (Dionex, Sunnyvale, CA). Protein identification and label-free quantitation was achieved as described previously^53^.

### Gel electrophoresis and Western blotting

Briefly, one dimensional electrophoresis was performed using NuPAGE 4–12% gradient Bis-Tris gels (Novex, LifeTechnologies) and MOPS SDS running buffer (Novex, Life Technologies) under a constant voltage of 90 V for 10 min and 180 V until the bromophenol blue front dye has reached the bottom of the gel. Transfer of proteins was performed overnight under a constant amperage of 0.11 A. For two-dimensional gel electrophoresis, cell lysates (8 M urea, 2 M thiourea, 4% (w/v) CHAPS, pH 8.5) were split in 100 μg aliquots, cleaned with the 2D-Clean-up kit (GE Healthcare Life Sciences, Pittsburgh, PA), re-solubilised in lysis buffer to a final concentration of 5 μg/μl, and loaded on 11 cm IEF gels with an immobilized nonlinear pH 3–11 gradient (Immobiline DryStrips, GE Healthcare) in an IPGphorIII apparatus (GE Healthcare). IPG strips were first rehydrated overnight in IPGphor strip holders (GE Healthcare) with 200 μl of the mixture of sample added with the destreak rehydratation buffer including 0.5% carrier ampholyte pH 3-11NL (GE Healthcare). The IEF protocol was as follows: 300 V gradient for 1 h; 1000 V gradient for 1.5 hr; 6000 V gradient for 2.5 hr; 6000 V step for 2 h. Temperature was set up at 20 °C. IPG strips were equilibrated for 10 min in an equilibration buffer (6 M urea, 50 mM Tris pH 8.8, 2% SDS, 38.5% glycerol) containing 65 mM DTT, and for 10 min in the same equilibration buffer containing 2% iodoacetamide. The second dimension was performed using a Criterion Dodeca Cell separation unit (Biorad, Hercules, CA) and precast criterion XT Bis-tris 4–12% SDS PAGE gels (Biorad). Temperature was set up at 20 °C. IPG strips were placed on the top of the precast gels, overlaid with 0.5% agarose in 2x running buffer containing bromophenol blue. 10 μl of Page ruler (Thermofisher scientific) was loaded in the dedicated well. Gels were run at 20 **°**C using 2X and 1X MES running buffer (Biorad) at the cathode and at the anode, respectively. Electrophoresis was conducted at 80 V during 10 minutes, then at 120 V until the bromophenol blue front dye has reached the bottom of the gel. After electrophoresis, proteins were transfered overnight at 4 °C with a Hoefer TE42 Standard Transfer Tank (Hoefer inc., Holliston, MA) on supported nitrocellulose membrane (GE Healthcare) under a constant voltage of 25 V. After transfer, membranes were washed on MilliQ water and colored with Ponceau Red for quality control of protein 2D separation.

### Microtubule and actin microfilament-pelleting assay

SKBr3 cells were scrapped in PBS, pelleted, and lysed in 1.5 volume of cell pellet of lysis buffer (MEM: 0.1 M MES, pH 6.9, 1 mM EGTA, 1 mM MgCl_2_ supplemented with 0.5% NP40, 1 mM DTT and protease/phosphatase inhibitor cocktails from Roche) for 45 min on ice. Cell lysates were centrifuged at 20800 g for 25 min at 4 °C. Cytosolic extracts were split in 100 μl aliquots that were incubated with either 10 μM paclitaxel (1% DMSO) for 30 min at 30 °C or 20 μM phalloidin (1% DMSO) for 30 min at 20 °C, respectively. After incubation, paclitaxel- or phalloidin-treated extracts were spun on a 100 μl 10% sucrose cushion in MEM 1% DMSO buffer containing either 10 μM paclitaxel or 20 μM phalloidin, for 30 min at 80000 g and 30 °C, or 150000 g and 20 °C, respectively. Supernatants were mixed with 35 μl of 4X Laemmli sample buffer and pellets were suspended in 135 μl of 1X Laemmli buffer, respectively. All samples were denatured at 95 °C for 10 min. The content of each supernatant and pellet was analyzed by Western blotting with 10 μl of sample loaded per gel well. Distribution of septins, tubulin and actin between supernatants and corresponding pellets was quantified using Image J software.

### Immunofluorescence and immunohistochemistry

Briefly, immunofluorescence was performed on cells cultured on collagen-coated (SKBr3, MCF7, HMLE, 184A1) or fibronectin-coated (HUVEC) coverslips fixed with 4% formaldehyde in 3% sucrose PBS and permeabilized with 0.2% Triton X-100. Images were acquired on a structured light ApoTome microscope (Zeiss, Münich, Germany) equipped with 63 × 1.4 plan ApoChromat objective and an AxioCam MRc5 camera. Immunohistochemistry was performed using the Discovery XT Automate from Roche (Tucson, AZ) and HRP/DAB staining. Tissue blocks in paraffin were cut in 3 μm thick slices with a microtome HM340E equipped with the slice transfer system (STS) (Microm Microtech, Brignais, France) and placed on FLEX IHC microscope slides (Dako). Slides were placed in the automate for 1) paraffin removal, 2) standard cell conditioning with Cell Conditioner 1 (CC1), 3) manual application of primary antibody (for 1 h for polyclonal pan septin 9 diluted at 1/2000 and monoclonal anti-septin 9_i2 diluted at 1/25; for 3 h for monoclonal anti-septin 9_i1 diluted at 1/25, at 37 °C), 4) application of one drop of OmniMap anti-Rb or anti-Rat HRP (Multimer HRP) for 16 min, 5) application of ChromoMap DAB Kit (RUO) for 8 min, 6) application of one drop of Hematoxylin II for 16 min, 7) application of one drop of Bluing Reagent for 4 min, 8) Washes. Primary antibodies were validated for IHC with expression controls (Supplementary Fig. S6) that were included in all IHC run as internal experimental standards. Slides were entirely scanned on a Nanozoomer 2.0HT scanner (Hamamatsu Photonics, Massy, France) and images were produced using the CaloPix 3.0.0 software (Tribvn, Chatillon, France). Histological examination and staining scoring of each slide was performed independently by two certified pathologists. An independent semi-quantitative analysis of each image using the RVB sampling tool of Adobe Photoshop software (RVB average on 12 sampling spots per image) was performed and led to very similar scoring results.

## Additional Information

**How to cite this article:** Verdier-Pinard, P. *et al*. Septin 9_i2 is downregulated in tumors, impairs cancer cell migration and alters subnuclear actin filaments. *Sci. Rep.* **7**, 44976; doi: 10.1038/srep44976 (2017).

**Publisher's note:** Springer Nature remains neutral with regard to jurisdictional claims in published maps and institutional affiliations.

## Supplementary Material

Supplementary Information

## Figures and Tables

**Table 1:** Expression of total Sept9 and of isoforms i1 and i2 in normal breast tissues and breast tumors.

Sample	Sept9		Sept9_i1		Sept9_i2	
	%^1^	Score^2^	%	Score	%	Score
Normal (N = 8)^3^	93	+/++	0	*nr*^4^	100	+++
Tumors (N = 40)	89	++	8	+	9	+/++
Luminal A (N = 10)	86	++	0	*nr*	0	*nr*
Luminal B (N = 10)	90	++	9	+	0	*nr*
HER2 + (N = 10)	87	++	6	+	0	*nr*
Basal (N = 10)	93	++	17	+	34	+/++

^1^Average percentage of positive epithelial/carcinoma cells.

^2^Score based on labelling intensity levels in positive cells.

^3^(N = number of independent tissue samples).

^4^Not relevant.
