# Supplementary material for: Septin 9_i2 is downregulated in tumors, impairs cancer cell migration and alters subnuclear actin filaments
Source: Sci Rep. 2017 Mar 24;7:44976. doi: 10.1038/srep44976 (PMC5364497; doi:10.1038/srep44976)
Supplement: Supplementary Information [file srep44976-s1.pdf]

Supplementary Information

Septin 9_i2 is downregulated in tumors, impairs cancer cell migration and alters subnuclear actin filaments

P. Verdier-Pinard, D. Salaun, H. Bouguenina, S. Shimada, M. Pophillat, S. Audebert, E. Agavnian, S. Coslet, E. Charafe-Jauffret, T. Tachibana and A. Badache

Supplementary Table S1. Fold enrichment of septins in GFP-pull downs (IP) on MCF7.GFP-Sept9_i1, _i2 or i5 cell line extracts compared to MCF7 cell line extracts. Fold enrichement values from two independent series of pull downs (#1 and #2) are presented.

**Septin group**	**Sept3**		**Sept7**		**Sept6**						**Sept2**			
**Detected septins**	**Sept9**		**Sept7**		**Sept11**		**Sept10**		**Sept8**		**Sept2**		**Sept5**	
IP	***#1***	***#2***	***#1***	***#2***	***#1***	***#2***	***#1***	***#2***	***#1***	***#2***	***#1***	***#2***	***#1***	***#2***
	**Fold enrichment**													
GFP-Sept9_i1	**66**	**114**	**51**	**88**	**26**	**95**	**93**	**56**	**3**	**96**	**47**	**14**	**10**	**12**
GFP-Sept9_i2	**15**	**69**	**9**	**38**	**6**	**52**	**5**	**18**	**2**	**46**	**6**	**12**	**3**	**10**
GFP-Sept9_i5	**39**	**48**	**77**	**74**	**42**	**66**	**77**	**25**	**3**	**76**	**46**	**12**	**18**	**7**

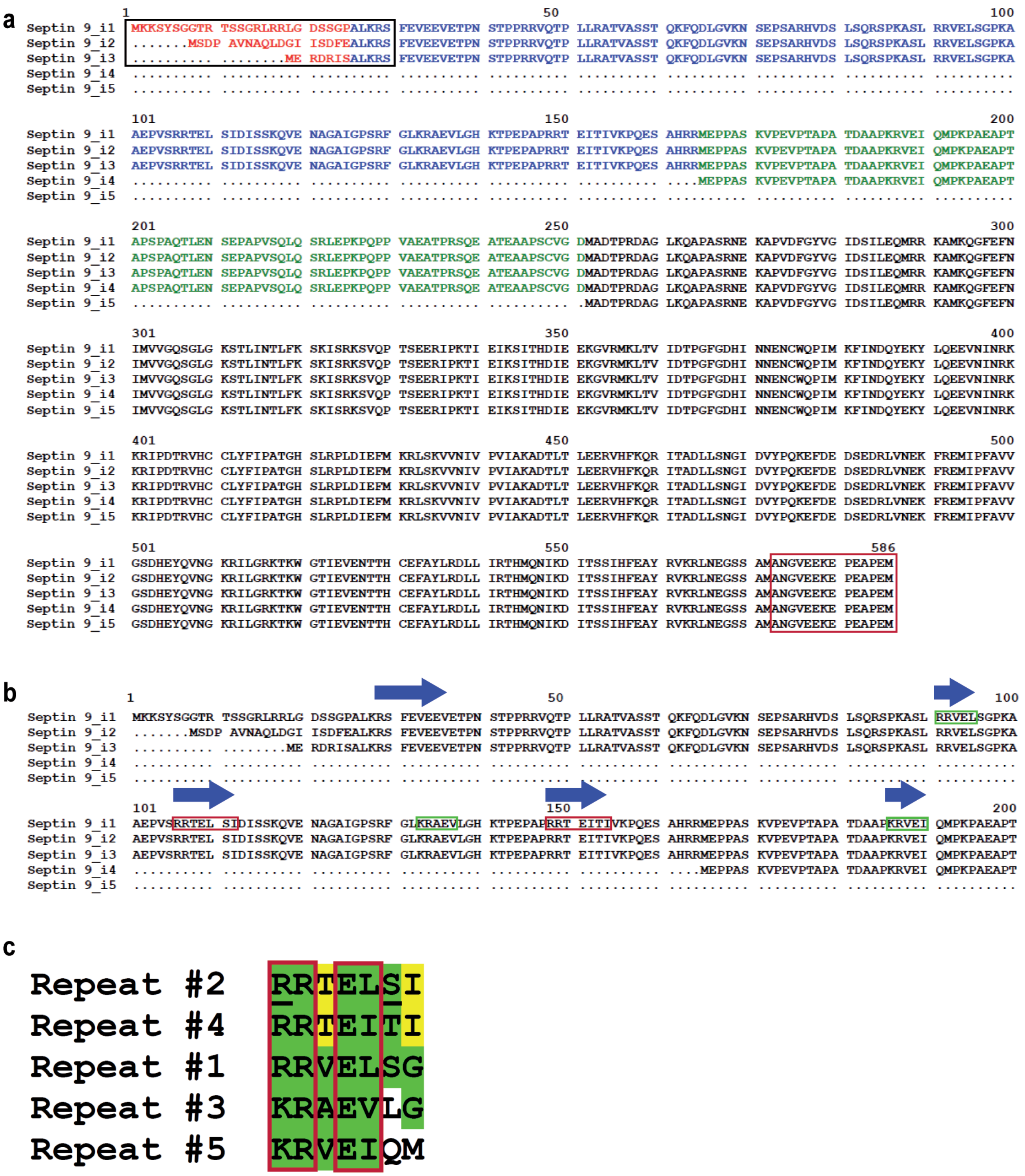

Supplementary Fig. S1. Septin isoform protein sequences. a) Sequence alignment of human Sept9 isoforms. In red, sequences specific to each long isoforms; in blue, sequence common to long isoforms; in green, N-terminal sequence of Sept9_i4, shared with the long isoforms; in black, corresponding sequence of Sept 9_i5, the shortest isoform. Sequences boxed with black and red frames correspond to the antigens used for producing the specific isoform-specific or pan-Sept9 antibodies, respectively. b) Sequence alignment of human Sept9 isoforms N-termini. Blue arrows indicate predicted β-sheet secondary structures (MeDor metaserver, http://www.vazymolo.org/MeDor/index.html). Green and red framed sequences correspond to short (#1, #3, #5) and long (#2, #4) imperfect repeats, that are aligned in c). Identical and similar amino-acid residues in short and long repeats or specific to long repeats are highlighted in green and yellow, respectively. Underlined residues in repeat #2 are mutated in HNA patients. Red framed sequences are conserved in all repeats.

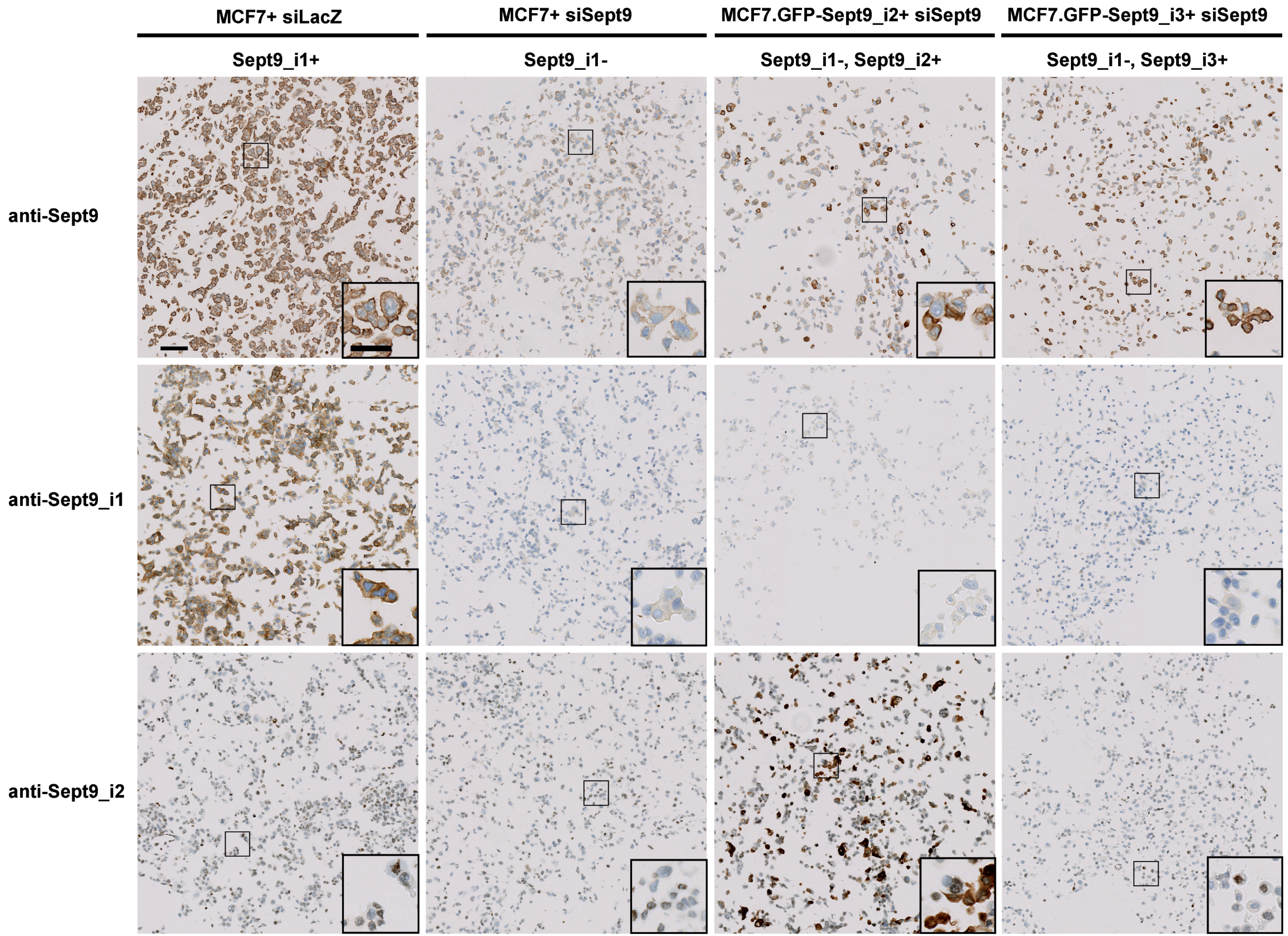

Supplementary Fig. S2. Validation of Sept9 antibodies for immunohistochemistry. MCF7 parental cells and stably expressing GFP-Sept9_i2 or _i3 cells were transfected either with a control siRNA (LacZ) or the Sept9#2 siRNA to deplete endogenous Sept9. At 72 h post transfection, cells were pelleted, fixed with formaldehyde and embedded in paraffin. Cell pellet sections were incubated with the polyclonal pan-Sept 9 antibody or the monoclonal antibodies against either Sept9_i1 or _i2. First column: parental MCF7 cells expressed only Sept9_i1 (see Figure 2b); second column: Sept9 siRNA knockdown cells have no or low Sept9 expression; third column: cells expressing only Sept9_i2; fourth column: cells expressing only Sept9_i3. The isoform specific antibodies against Sept9_i1 or _i2 are specific for Sept9_i1 or _i2, respectively, with no cross-reaction with other Sept9 isoforms. The antibody against Sept9_i2, showed non-specific nuclear labeling in all tested samples, clearly cross-reacting with a non-Sept9 nuclear antigen. Inserts correspond to zoomed boxed areas. Black horizontal bars represent 100 μm and 60 μm in images and zoom inserts, respectively.

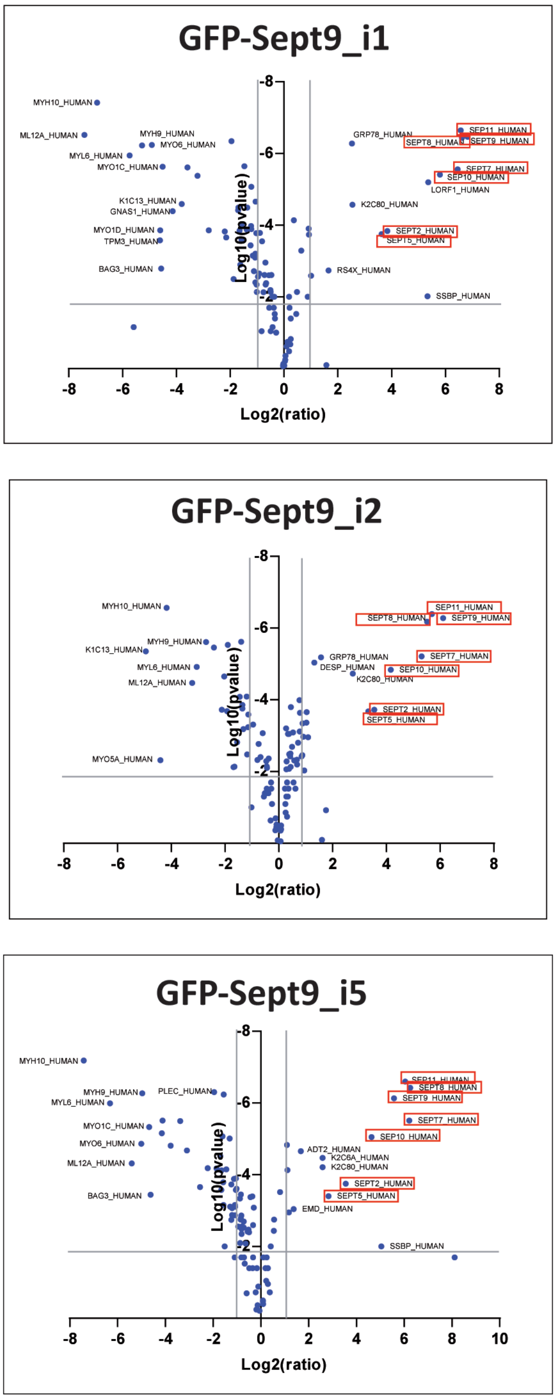

Supplementary Fig. S3. Vulcano plots of proteins specifically enriched in GFP-pull down #2 from extracts of MCF7 cells stably expressing GFP-Sept9_i1, _i2 or _i5. Thresholds (thin grey lines) for ratio and p-values were 2 and 0.012, and plotted as Log2 and Log10, respectively. Proteins that were specifically enriched are in the upper right corner and are boxed in red when present in both pull-downs #1 and #2. Only septins were found to fit these criteria.

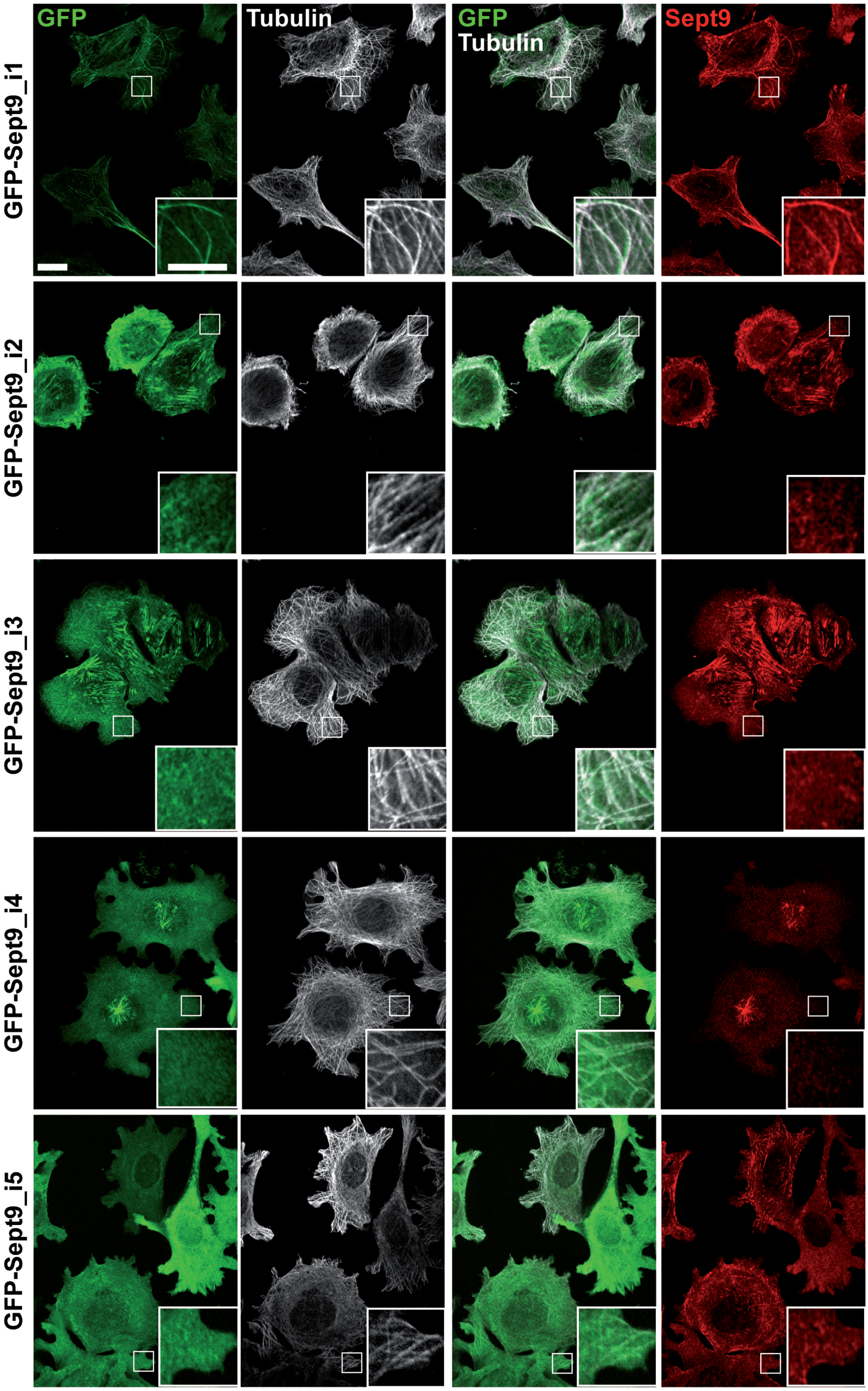

Supplementary Fig. S4. Co-localization of Sept9_i1 with microtubules. Labeling of microtubules and GFP-Sept9 isoforms stably expressed in MCF7 cells showed that only Sept9_i1 was associated with microtubules. Framed regions are zoomed at the bottom of each image. White bars correspond to 10 μm and 5 μm in image and zoomed regions, respectively.

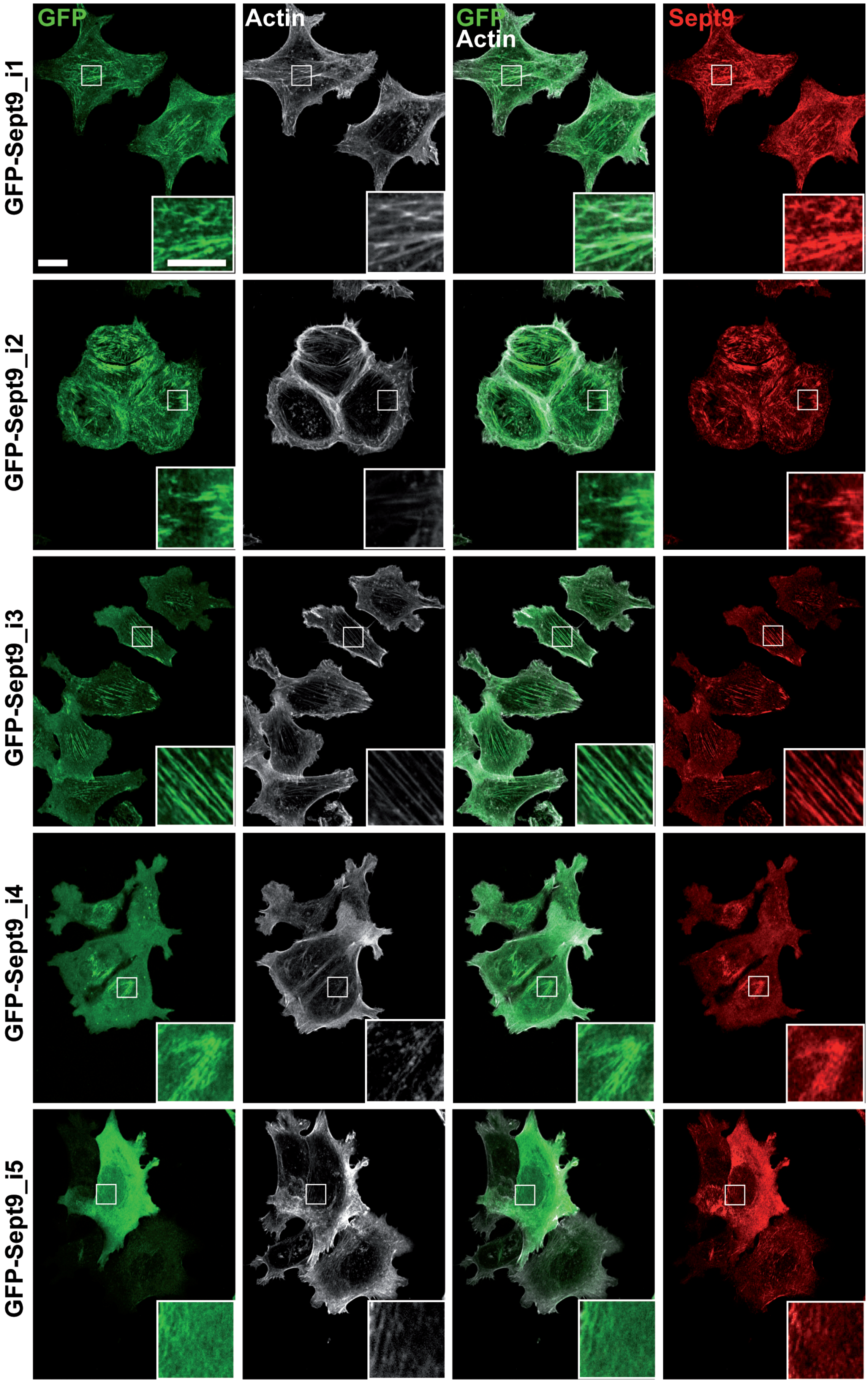

Supplementary Fig. S5. Absence of co-localization of Sept9_i5 with microfilaments. Labeling of microfilaments and GFP-Sept9 isoforms stably expressed in MCF7 cells showed that only Sept9_i5 was not associated with actin filaments. Framed regions are zoomed at the bottom of each image. White bars correspond to 10 µm and 5 µm in image and zoomed regions, respectively.

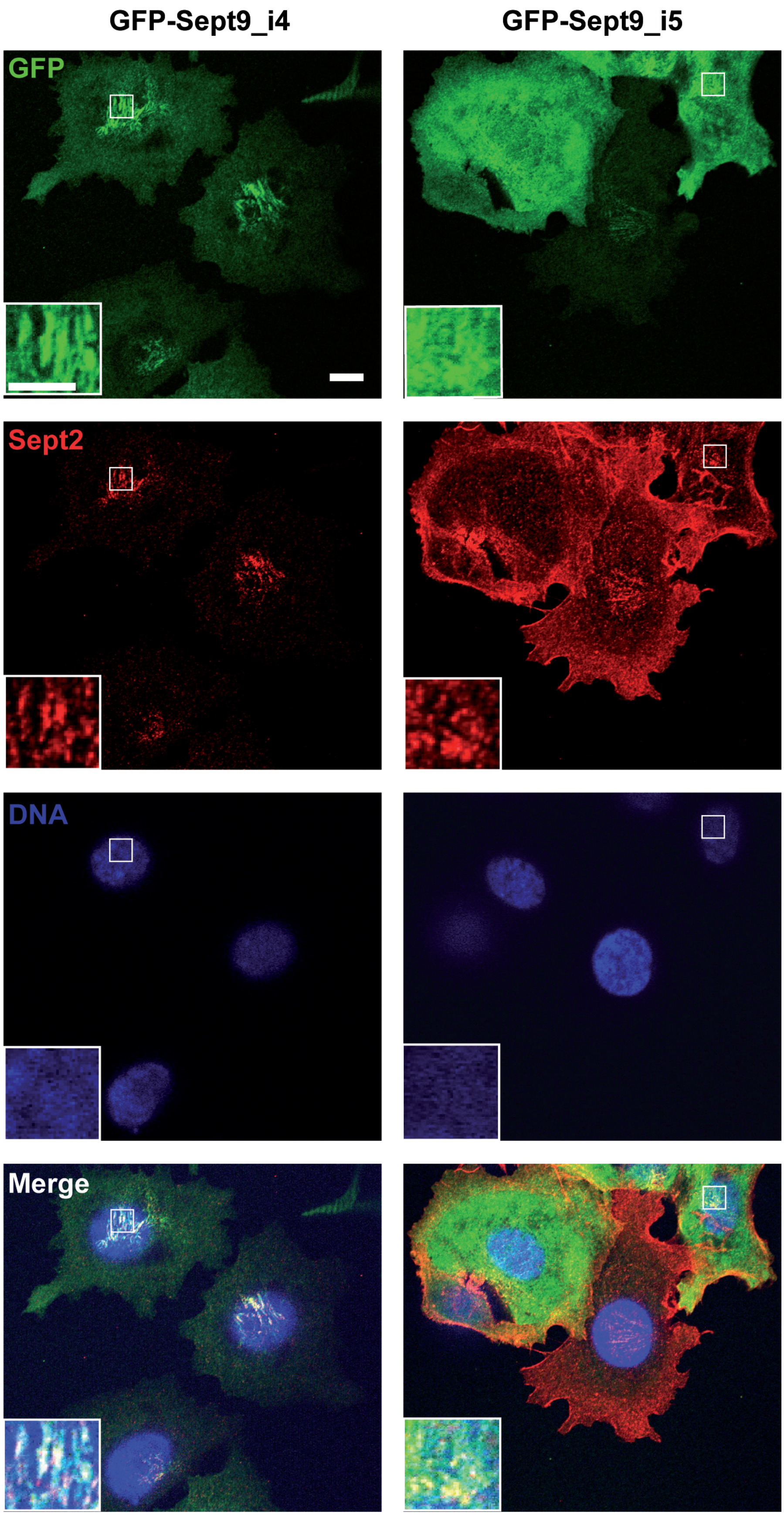

Supplementary Fig. S6. Co-localization of Sept9_i4, but not of Sept9_i5 with Sept2-containing filaments.
Labeling of Sept2 in MCF-7 cells stably expressing either GFP-Sept9_i4 or GFP-Sept9_i5 showed that Sept9_i4 co-localized with Sept2 in short filaments concentrated under the nucleus, whereas overexpressed Sept9_i5 and endogenous Sept2 displayed a diffuse cytoplasmic distribution. Framed regions are zoomed at the bottom of each image.
White bars correspond to 10 μm and 5 μm in image and zoomed regions, respectively.

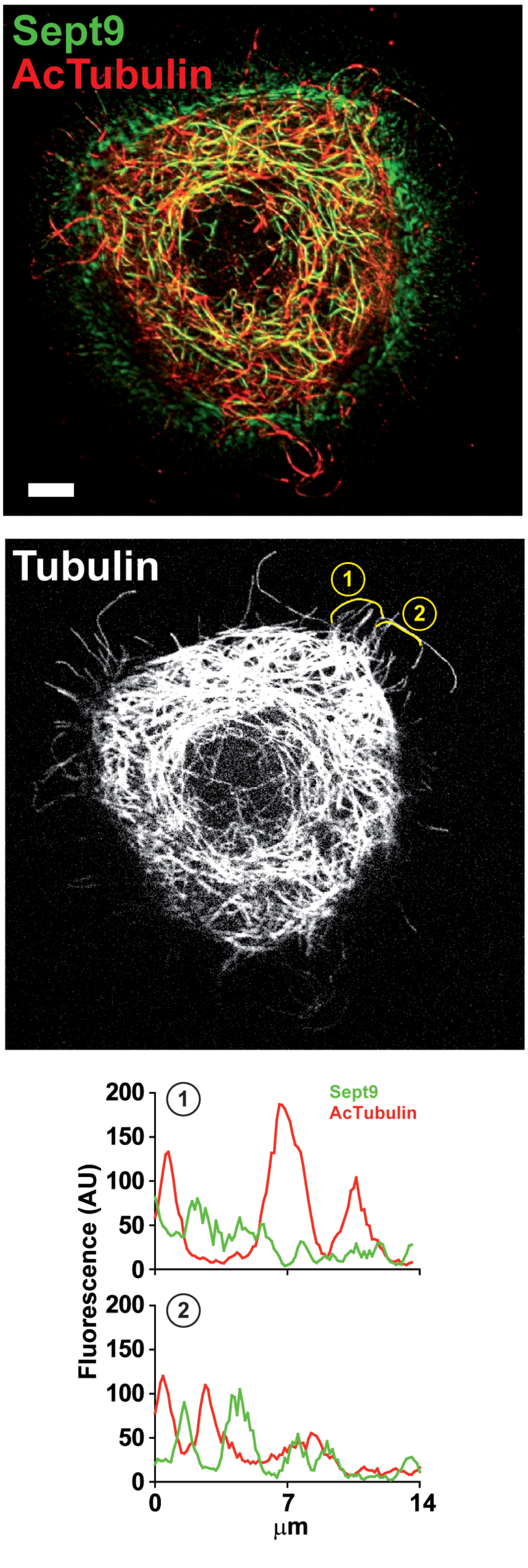

Supplementary Fig. S7. Alternative co-localization of Sept9 and acetylated tubulin with microtubules.
Labeling of Sept9 and acetylated tubulin in SKBr3 cells (uncropped and unzoomed image of Fig. 8c) showed that Sept9 was co-localizing with a subpopulation of microtubules that were acetylated. Fluorescence intensity profiles along weakly labeled peripheral microtubules revealed that Sept9 and acetylated tubulin tended to label microtubules in an alternative complementary pattern (as depicted in profiles from microtubule sections 1 and 2 marked in yellow on image). Fluorescence intensity profiles were generated using the free line drawing and plot profile tools of ImageJ software (NIH). Fluorescence intensities of tubulin labeling was subtracted from fluorescence intensities of Sept9 or acetyl tubulin labeling. White bar corresponds to 10 µm.

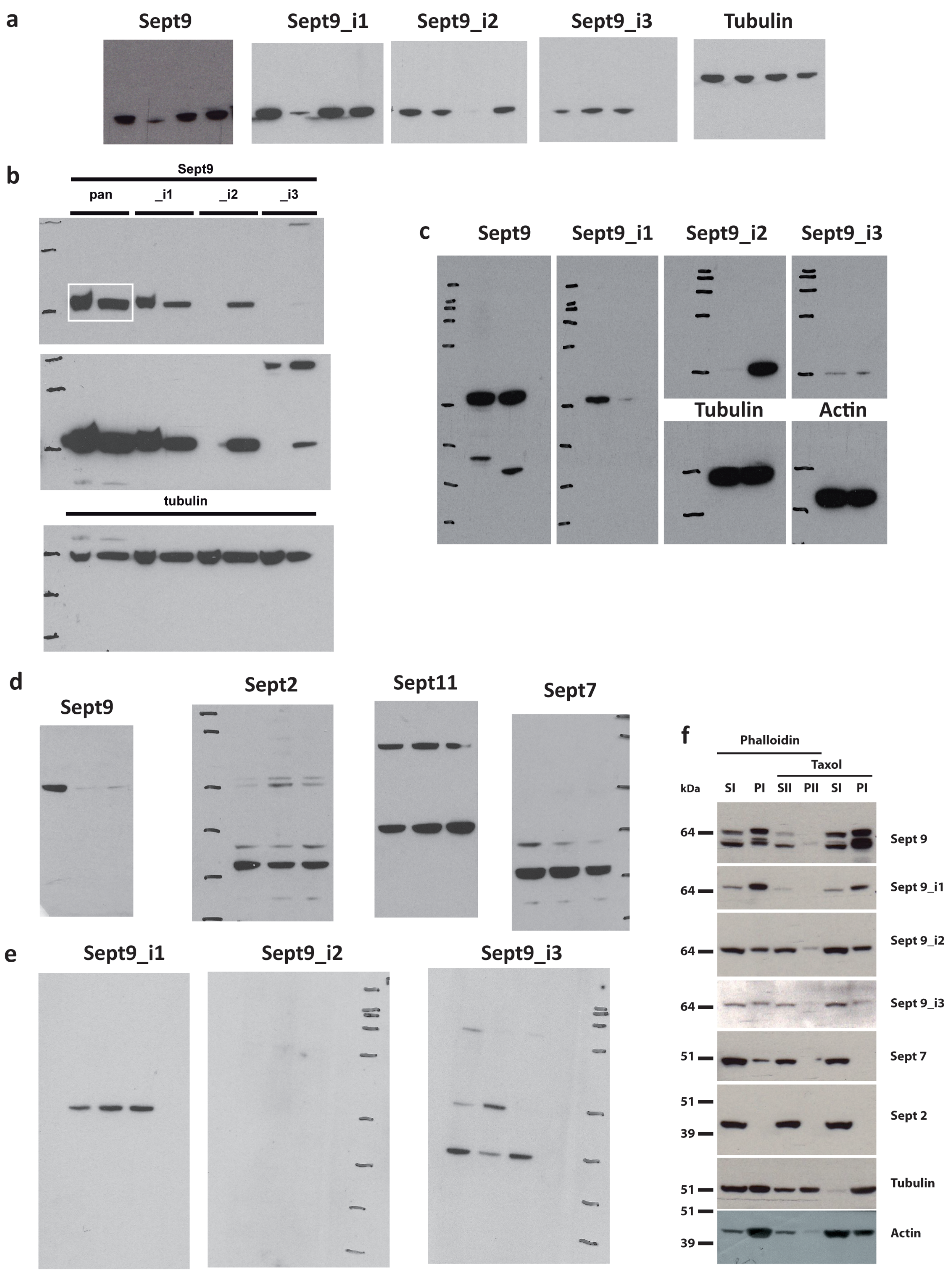

Supplementary Fig. S8. Uncropped Western blots used in the figures. a) Fig. 2a. b) Fig 2b, first lane for each antibody was for MCF7 cell extracts, image was cropped for each antibody as shown with the white box and mirrored vertically to get the SKBr3 extracts in first lane in Fig. 2b; the middle panel is a longer exposure time to auto-radiographic film that was used to obtain a signal for Sept9_i3 comparable to those of Sept9_i1 and _i2. c) Fig. 2c. d) Fig. 5a. e) Fig. 8b. f) Fig. 9a, the first two, and the last two lanes were used.
